# Multi-Modal Physics-Informed Neural Network for Single-Track Geometry Prediction in Powder-Bed Arc Additive Manufacturing of 316L Stainless Steel

**DOI:** 10.3390/ma19163454

**Published:** 2026-08-14

**Authors:** Arif Balcı

**Affiliations:** Department of Mechanical Engineering, Faculty of Engineering and Architecture, Kafkas University, Kars 36100, Türkiye; arifbalci@kafkas.edu.tr

**Keywords:** powder-bed arc additive manufacturing, PBAAM, 316L stainless steel, physics-informed neural network, PINN, single-track morphology, Ayrton voltage, multi-modal learning, bootstrap confidence interval, pure speed extrapolation

## Abstract

This study presents a methodology for predicting the geometric features of single tracks of 316L stainless steel produced by Powder-Bed Arc Additive Manufacturing (PBAAM) from four independent process parameters using a multi-modal Physics-Informed Neural Network (PINN). PBAAM shares the same powder-deposition and layering scheme as Laser Powder Bed Fusion (LPBF) but uses a low-current micro-TIG arc rather than a laser as the heat source. A multi-task PINN architecture was developed that simultaneously predicts five geometric features measured from two imaging modalities (top-view and side-view arc), namely the arc core diameter (D_q_), the arc cone angle (α_c_), the heat-affected zone width (w_HAZ_), the track core width (d_core_) and the areal equivalent track width (w_iz_), from four input parameters (arc current, traverse speed, work angle and working distance). The model was assessed on a full-factorial training matrix of 36 experiments and on four pure speed extrapolation experiments above the training range. A composite quality score filter classified 23 of the training experiments as stable and 13 as unstable. On the pure validation set, the mean absolute percentage error (MAPE) was 4.25% (95% confidence interval 0.91–8.49) for the arc core diameter, 6.29% (5.07–7.59) for the arc cone angle, 8.06% (6.08–9.82) for the heat-affected zone width, and 17.02% (10.77–21.62) for the track core width. Classical regression baselines attain comparable aggregate errors on this narrowly distributed validation set; the distinguishing property of the proposed model is the joint, physically ordered prediction of all five outputs. The Ayrton voltage sub-module of the model converged to U(I) = 11.33 + 97.13/I without any direct voltage measurement, purely through the physics loss term; this function is consistent with the order of magnitude expected from the physics of low-current TIG arcs. The results indicate that physics-informed learning can be applied to the PBAAM process parameter space under small-sample conditions. This capability is demonstrated for 316L stainless steel, for the micro-TIG electrode configuration and the process window investigated here, for single tracks rather than multi-layer builds, and against a validation set of four experiments varying in a single direction.

## 1. Introduction

Metallic additive manufacturing (AM) has gained a significant position over the past two decades in the aerospace, biomedical and energy sectors thanks to its ability to produce geometrically complex parts in a single step. Within the powder-bed fusion (PBF) family, laser powder bed fusion (LPBF) has become the most widely adopted method at industrial scale [1]; however, the high-power laser source and narrow process window considerably increase capital and operating costs.

These constraints have raised the importance of alternative configurations that preserve the powder-bed deposition logic of LPBF but replace the heat source with low-cost and widely accessible arc processes. In this respect, plasma powder-fed additive manufacturing (PPA-AM) [2], the PBF-TIG approach using a tungsten inert gas (TIG) arc as the heat source over a powder bed [3] and powder-bed arc additive manufacturing (PAAM) [4] have been systematically evaluated in the literature. Horbenko and Zvorykin [5] analytically discussed the feasibility of using a TIG arc as a laser alternative for powder sintering and assessed the efficiency and cost prospects of this configuration relative to laser-based processes.

Powder-bed arc additive manufacturing (PBAAM) is the subset of this process family that most closely resembles LPBF in geometric terms: it uses the same powder-deposition scheme, the same layering logic and the same methodological infrastructure that was developed for the LPBF process window. The only fundamental difference is that the heat source is a low-current micro-TIG arc instead of a high-power laser beam. In the authors’ previous work [6], the PBAAM process was developed in-house and the main effects of process parameters (current, traverse speed, layer thickness and overlap distance) on melt-pool width and track continuity were identified for single tracks of 316L stainless steel. Çelikel and Balcı [7], using a fixed 0.6 mm tungsten electrode diameter on the same system, mapped Continuous-Stable, Transitional and Fragmented morphological regimes across 17 different process conditions via image-based topological analysis (connected-component count *N* and width coefficient of variation CV).

Although these two studies took an important step in macro-experimentally mapping the PBAAM process window, a predictive model that jointly captures, under four independent process parameters (current, speed, work angle, working distance), the five geometric features extracted from two different imaging modalities (top-view and arc) has not yet been presented. A single model that can jointly predict multiple outputs of the PBAAM process window (track width, core width, heat-affected zone and arc geometry) is important both for process optimisation and for systematic powder-bed parameter selection at the component scale.

The engineering value of these geometric features lies in the metallurgical outcomes they govern. Single-track width and the associated melt-pool dimensions set the hatch spacing and layer thickness at which neighbouring tracks and successive layers overlap sufficiently to fuse. Where the overlap is inadequate, unmelted powder remains trapped between deposits and lack-of-fusion defects arise. Predicting these features therefore provides a route to anticipating fusion quality, inter-layer bonding and defect formation before deposition. Porosity develops [8] a defect population that is among the most detrimental to fatigue performance. Track width and its variability along the deposit likewise govern the transition between continuous and balling morphologies, and the boundaries of the usable process window are conventionally drawn from precisely these two criteria [9]. The heat-affected zone width carries different information. It measures the thermal footprint of the arc on the surrounding powder bed and therefore indicates how much loose powder is sintered or partially bonded at the periphery of the track, which has a bearing on powder reuse, on surface roughness and on the energy available to the subsequent deposit. The arc-side features describe how that thermal footprint is delivered, since the diameter and the cone angle of the plasma column determine whether the energy arrives concentrated or diffuse, and this in turn shapes melt-pool convection and the resulting solidification structure [10,11]. Predicting these features from the process parameters is therefore not purely geometric.

The training matrix is a complete four-factor factorial design at three levels of arc current, three levels of traverse speed, two levels of work angle and two levels of working distance, which yields 3 × 3 × 2 × 2 = 36 conditions. Because the design is full rather than fractional, no effect is aliased with any other, and all two-factor and higher-order interactions among the four parameters are estimable at the sampled levels. The three-level treatment of current and speed additionally permits curvature in these two factors to be resolved, which matters because the thermal response to both is expected to be nonlinear. Work angle and working distance are sampled at two levels and therefore support linear effects and interactions only. The parameter bounds were fixed by the operating envelope of the process rather than chosen freely. Below approximately 15 A the arc does not sustain continuous melting of the powder bed, and above 25 A the bed is displaced by the arc pressure. Regarding repeatability, no condition was replicated, so the dataset does not support a direct estimate of experiment-to-experiment variance. This is stated as a limitation in Section 5. What can be quantified from the present measurements is the variability of the track itself. The coefficient of variation in the local width profile averages 0.217 over the training set and ranges from 0.115 to 0.406, which reflects genuine morphological instability rather than measurement noise and is the basis of the quality classification described in Section 2.5.

The principal obstacle in closing this gap is the structural tension between small-sample datasets and multi-output modelling. While systematic single-track studies for LPBF can accumulate large numbers of tracks owing to the speed of the process [9,12], single-track experiments in PBAAM are costly in time and material; the dataset therefore typically remains on the order of 20–40 experiments. At this sample size, purely data-driven deep-learning approaches (e.g., deep fully connected networks, gradient-boosted trees) tend to overfit and fail to generalise outside the training range [13].

Physics-informed neural networks (PINNs) [14] have emerged as a promising alternative in such low-data regimes. PINNs include an additional loss term that enforces compatibility of network outputs with a physical constraint (a partial differential equation, conservation law, monotonicity rule, etc.), thereby simultaneously providing data fit and physical consistency [15,16]. PINN applications for metallic AM processes have expanded rapidly in recent years: Zhu et al. [17] successfully modelled melt-pool temperature and fluid dynamics in LPBF; Xie et al. [18] modelled 3D temperature fields in direct energy deposition (DED); Zobeiry and Humfeld [19] applied PINN to heat transfer in advanced manufacturing processes. Hosseini et al. [20] demonstrated that a parametric physics-informed neural network can predict temperature fields and melt-pool sizes for single-track LPBF with a mean absolute error below 5% relative to the finite element reference solution; Sajadi et al. [13] reported that PINN preserves sustainable performance under distribution shift even in an online-learning regime.

In the most recent comprehensive review, Faegh et al. [21] showed that PIML applications in AM fall into three principal categories: (i) approaches that add physics constraints as soft penalty terms in the classical loss function, (ii) approaches that embed physics-based sub-modules in the network architecture, and (iii) multi-modal learning. The review also emphasised that multi-output PINN architectures exhibit statistically superior performance over single-output models under small-sample conditions.

Within this taxonomy, the framework proposed in the present study draws on all three categories, and it is useful to state at the outset which of them carry the physical information. The approach is not a classical PINN in the sense of Raissi et al. [13], where the residual of a governing partial differential equation is evaluated at collocation points and driven towards zero. No such residual is formulated here. Physical knowledge enters through the first two categories in distinct ways: four soft penalty terms are appended to the data-fit loss, and one physical sub-model is embedded within the network itself, while the third category describes the multi-output architecture. The four penalty terms are a Rykalin-type heat-conduction scaling residual, which ties the predicted equivalent track width to the effective energy input per unit length; a geometric ordering residual enforcing the physically required relations among the heat-affected zone, the melted track and the arc dimensions; a monotonicity residual acting on the partial derivatives of the network outputs with respect to the arc current; and a box residual restricting the arc voltage to the interval that is physically admissible for a low-current arc. The embedded sub-model is the Ayrton voltage relation U(I) = U_0_ + k/I, whose two parameters are learned jointly with the network weights and without any direct voltage measurement. It is not itself a residual but enters the heat-conduction residual through the effective energy term and is bounded by the box residual. Every residual is imposed as a soft penalty rather than as a hard constraint, so that the network retains the freedom to depart from an imposed relation where the experimental evidence requires it. The explicit mathematical form of each residual, together with the assumptions on which it rests, is given in Section 2.7.

Against this background the specific novelty of the present contribution can be stated precisely. Neural and convolutional models of melt-pool and bead geometry are by now well established, and physics-informed variants have been reported for temperature fields and for porosity. What has not been reported is a single model that predicts, from four process parameters alone, a set of five geometric features drawn from two different imaging modalities, while enforcing the ordering relations that must hold between features belonging to different modalities, and while recovering a process quantity that was never instrumented. The novelty therefore lies in three specific choices rather than in the use of a physics-informed loss as such: the cross-modal output set, the cross-modal consistency constraints that couple its members, and the embedded electrical sub-model whose parameters are identified from geometry rather than from electrical measurement. The process itself is a further point of difference, since PBAAM has not previously been treated with a physics-informed model.

However, the majority of the existing PINN-AM literature focuses on LPBF or DED processes; no directly adapted PINN application for arc-based powder-bed processes has been reported. This gap is connected to the fact that arc physics, particularly in the low-current TIG regime [22], exhibits a different multi-physics character from laser-driven processes: the Ayrton voltage–current relation [23], the angular spreading of the plasma column [10], and the powder-bed deposition dynamics [11]. Therefore, the development of a PINN architecture specifically for the PBAAM process constitutes a methodologically distinct contribution, both by integrating the unique components of arc physics (Ayrton voltage, arc cone angle) into the model and by preserving geometric consistency among outputs under small-sample conditions.

The principal contributions of this study can be summarised under three headings. First, a multi-modal PINN architecture (top-view and arc images as target modalities, with cross-section imaging for complementary verification) is defined for the PBAAM process for the first time. Second, the areal definition of the equivalent track width (w_iz_ = A_iz_/L_iz_) eliminates the systematic bias introduced by the classical maximum-width definition under unstable and fragmented tracks. Third, the Ayrton voltage sub-module is learned indirectly through the physics loss term without any direct voltage measurement, providing a concrete instance of the indirect parameter estimation capability of physics-informed learning [14,24]. The study further tests the model’s generalisation capability outside the training range through pure speed extrapolation, and reports statistically defensible performance values for small-sample conditions through a 1000-resample bootstrap analysis.

## 2. Materials and Methods

This study presents a methodology for predicting the geometric features of single tracks of 316L stainless steel produced by Powder-Bed Arc Additive Manufacturing (PBAAM) from four independent process parameters using a multi-modal Physics-Informed Neural Network (PINN). PBAAM shares the powder-deposition and layering scheme of Laser Powder Bed Fusion (LPBF) but uses a low-current micro-TIG arc rather than a laser beam as the heat source. This structural similarity makes it possible to adapt directly to PBAAM the melt-pool physics established for LPBF [11], the analytical process maps [25], the melt-pool overlap models [8] and the powder-bed thermal-conductivity models [26]. The proposed approach combines experimental image-based data with a Rykalin-type heat-conduction scaling and the electrical behaviour of the TIG arc (the Ayrton voltage–current relation), aiming to achieve high generalisation capability with limited data.

This section is organised in five blocks. Section 2.1 describes the experimental apparatus, the feedstock and the substrate preparation. Section 2.2 sets out the design of the experiments and the separation between the training matrix and the pure extrapolation set. Section 2.3, Section 2.4 and Section 2.5 cover data acquisition, that is the image processing chain, the extraction of the five geometric features and the quality classification that determines which measurements enter the loss for which output. Section 2.6 describes the machine-learning methodology, comprising the architecture, the training procedure and the preprocessing of inputs and targets. Section 2.7 gives the physics formulation, and Section 2.8 the bootstrap procedure used to obtain confidence intervals. The validation strategy is stated in Section 2.2.2 and its results are reported in Section 3.4, Section 3.8 and Section 3.10.

### 2.1. PBAAM System

**Figure 1:**
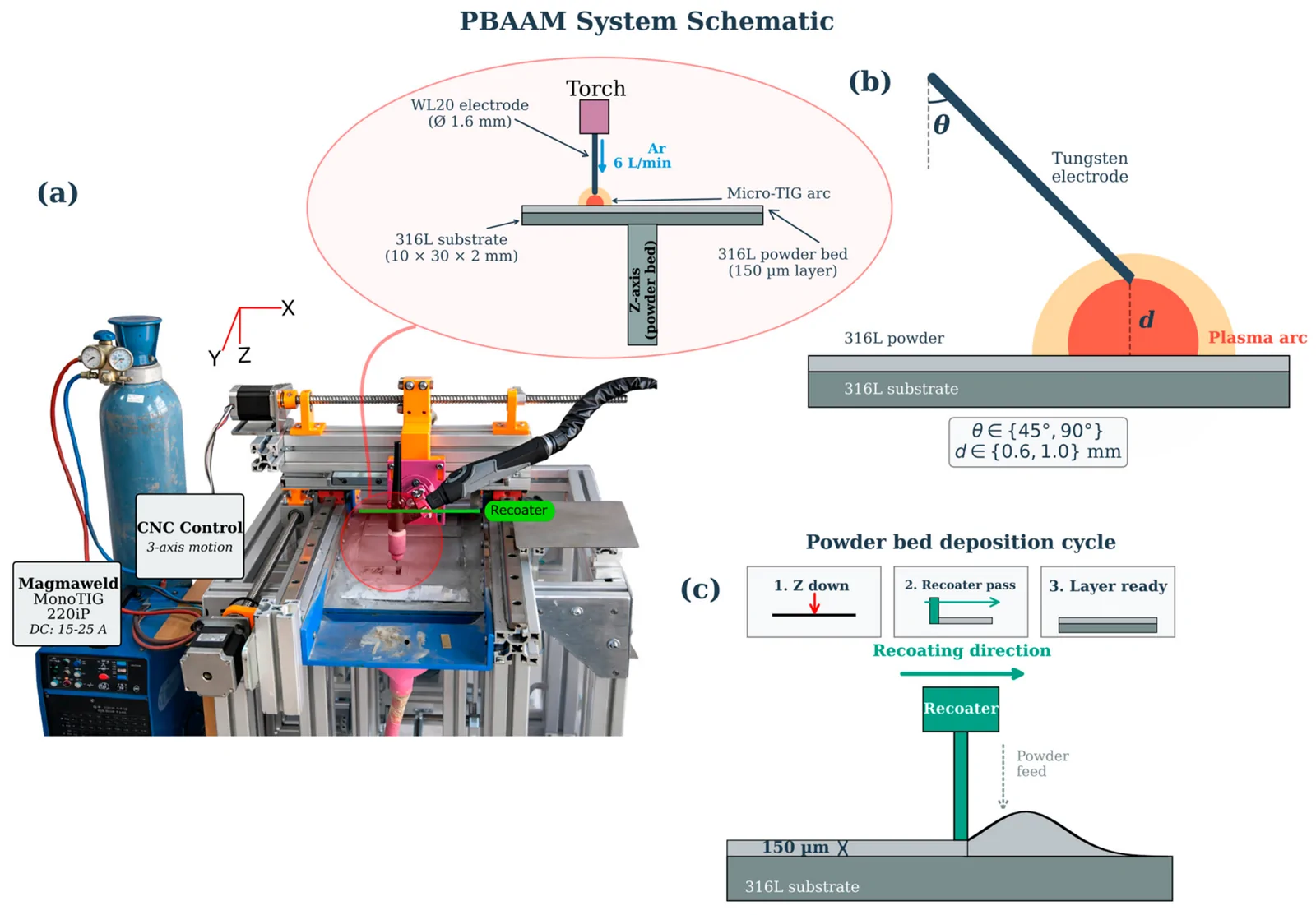
Schematic of the PBAAM system: (**a**) overall layout and main subsystems (Magmaweld MonoTIG 220iP power source, sigma-profile based three-axis CNC platform, elastomeric recoater, gas lens); (**b**) close-up of the electrode region (WL20 electrode, arc gap d, work angle θ); (**c**) powder-deposition mechanism (recoater pass forming a 150 μm fixed layer).

The PBAAM system comprises three principal subsystems: (i) a commercial micro-TIG arc welding unit serving as the heat source, (ii) a powder-bed deposition and layering mechanism, and (iii) a numerically controlled (CNC) positioning system providing axial motion and process control. The system shares the same powder-deposition and layering logic as LPBF; the only structural difference is that the heat source is a low-current TIG arc rather than a high-energy laser beam. The general layout and main components of the system are shown schematically in Figure 1. The system has been developed at Kafkas University and was also used in a previous study [6].

#### 2.1.1. Heat Source and Electrode Configuration

The heat source was a Magmaweld MonoTIG 220iP AC/DC water-cooled (Magmaweld, Manisa, Turkey) welding machine. The unit was operated in direct-current (DC) mode within a low current range of 15–25 A. This range corresponds to the lower bound of the operating window of conventional manual TIG welding and is referred to here as the micro-TIG regime. In this regime, the arc is constricted within a small area (≈1–2 mm in diameter) and produces a more diffuse, lower power-density heat distribution than is typical of LPBF processes.

The electrode used was a 1.6 mm diameter tungsten rod doped with 2% lanthanum oxide kitWELD WL20. The electrode tip was conically ground in the manner standard for TIG welding. The electrode-to-powder-bed working distance (arc gap) is one of the process parameters investigated at two levels, namely 0.6 and 1.0 mm (see Section 2.2). The work angle of the electrode with respect to the powder-bed surface was varied at two levels: 45° and 90°.

High-purity argon was supplied as the shielding gas at a constant flow rate of 6 L/min through a gas lens surrounding the electrode. The argon flow was maintained continuously throughout the experiments to stabilise the arc and to protect the molten pool surface from atmospheric oxidation.

#### 2.1.2. Powder Bed and Deposition Mechanism

Commercial gas-atomised 316L stainless-steel powder of spherical morphology was used as the feedstock material (Jiangsu Vilory Advanced Materials Technology Co., Ltd., Xuzhou, China; batch no. 202306-PRD-67). The powder has a particle-size range of 15–53 μm with size distribution characterised by D_10_ = 18.7 μm, D_50_ = 31.1 μm and D_90_ = 51.7 μm. According to the supplier’s quality certificate, the chemical composition (wt.%) was Cr: 16.83, Ni: 10.93, Mo: 2.27, Mn: 0.67, Si: 0.66, P: 0.033, C: 0.0080, S: 0.005, Fe: balance, with oxygen and nitrogen contents of 510 ppm and 936 ppm, respectively. This size range is consistent with the standard powder size distribution widely employed in industrial LPBF and is suitable for forming a fluid molten pool under arc-based melting conditions. The same powder batch has been characterised in detail in the author’s previous study [6].

The deposition system consists of a mechanically synchronised recoater unit with an elastomeric rubber tip mounted on the same sigma profile as the electrode. This type of compliant blade, which is common in LPBF systems, passes over the powder bed during the Y-axis traverse and forms a new layer of fixed thickness. The advantage of the elastomeric profile is that it tolerates surface irregularities better than rigid metal blades and does not produce localised compaction of the powder bed. The nominal layer thickness was kept constant at 150 μm for all experiments.

The substrates were 316L stainless-steel plates of dimensions 10 × 30 × 2 mm. Substrate surfaces were mechanically polished and cleaned with ethanol prior to the experiments to ensure proper wetting behaviour of the molten pool. Before each experiment, the recoater was positioned to make a single Y-direction pass and deposited a single layer of 316L powder of nominal thickness on the substrate.

#### 2.1.3. Motion Control and Process Automation

The system is integrated on a custom-built three-axis CNC platform built from aluminium sigma profiles. Stepper-motor-driven slides provide precise positioning and a constant traverse speed along the X, Y and Z axes. The electrode and recoater move in the X and Y directions on the upper sigma profile, while the powder bed moves downwards in the Z direction by the nominal layer thickness after each deposition pass. The traverse speed was varied as an experimental parameter at three levels in the range 0.25–0.75 mm/s.

At the start of each track, the arc was struck after the recoater pass had been completed, with the electrode moving at a constant speed in the X direction; at the end of the track the current was ramped down progressively to extinguish the arc in a controlled manner. This procedure was adopted to minimise transient irregularities at the start- and end-of-track regions in the single-track experiments.

#### 2.1.4. Position of PBAAM Within the AM Process Family

The PBAAM process can be positioned as a sub-class of the wider arc-based additive manufacturing family. Table 1 summarises the principal applications in this process family. The PBF-TIG process [3] is the first powder-bed approach to use the TIG arc as a heat source, but it operates in the macro-scale power and speed range (50–70 A, 37–57 mm/min). Plasma Arc Additive Manufacturing (PAAM) [4] combines wire feeding with a plasma arc, while Plasma Powder-fed Additive Manufacturing (PPA-AM) [2] uses a powder-fed plasma transfer arc. Horbenko and Zvorykin [5] systematically evaluated the use of the TIG arc as a laser alternative for powder sintering. The PBAAM process investigated here corresponds to a configuration that adapts the PBF-TIG framework proposed by Moreira et al. [3] to the micro-TIG scale (15–25 A, 0.25–0.75 mm/s) and to 316L stainless steel.

The ambient conditions of the laboratory were monitored but not actively controlled. Room temperature varied between 20 and 24 °C and relative humidity between 38 and 43 per cent across the campaign. Deposition was carried out in open atmosphere rather than in a sealed chamber, with the melt pool and the surrounding heated region protected by high-purity argon delivered through the gas lens at a constant 6 L/min as described above. The powder was used in the as-received condition and was not dried before use. The sealed container, which contained silica gel desiccant, was opened for the first time immediately before the powder was transferred to the hopper. Each experiment was performed on a separate substrate plate, so no heat accumulated in the substrate from one condition to the next and no waiting interval for substrate cooling was required between experiments. These conditions are reported for completeness. Because temperature and humidity were not varied deliberately, their residual variation is not represented in the model and forms part of the unmodelled scatter discussed in Section 5.

### 2.2. Experimental Design

Four principal process parameters governing the single-track behaviour of the PBAAM process were varied systematically: arc current (I, A), traverse speed (v, mm/s), electrode work angle (θ, °) and electrode-to-powder-bed working distance (d, mm). These four parameters are identified in the literature as the primary variables for arc-based powder-bed additive manufacturing studies due to their effects on melt-pool geometry, arc stability and energy transfer [3]. Other process parameters, including layer thickness (150 μm), gas flow rate (6 L/min) and electrode diameter (1.6 mm), were held constant in all experiments so that the effects of the four primary variables could be evaluated independently.

#### 2.2.1. Training Set: Full-Factorial Design

A full-factorial experimental design was used for training the PINN model. The arc current was varied at three levels (15, 20 and 25 A), the traverse speed at three levels (0.25, 0.50 and 0.75 mm/s), the work angle at two levels (45° and 90°), and the working distance at two levels (0.6 and 1.0 mm). This configuration produces 3 × 3 × 2 × 2 = 36 distinct combinations of process parameters. The full-factorial design was preferred because it allows unbiased sampling of the main effects and second-order interactions among the parameters. The lower current bound (15 A) was identified in our previous work as the threshold value at which continuous melting could be sustained; the upper bound (25 A) corresponds to the level at which the micro-TIG regime is preserved and no thermal-accumulation-driven geometric overgrowth is observed [7].

#### 2.2.2. Pure Validation Set: Speed Extrapolation

Four additional experiments outside the training range were performed to assess the generalisation capability of the PINN model. In these experiments the arc current was fixed at 25 A, the work angle at 45° and the working distance at 0.6 mm; only the traverse speed was extended above the training upper bound (0.75 mm/s), to 1.00, 1.25, 1.50 and 1.75 mm/s, respectively.

This strategy corresponds to the validation approach known in the literature as pure speed extrapolation. Conventional k-fold cross-validation samples the training and test sets from the same distribution and therefore measures only in-distribution generalisation capability. By contrast, extrapolation outside the training range tests directly whether the model can produce physics-consistent predictions under out-of-distribution conditions [15,19].

It should be emphasised that these four experiments are independent of model development in every respect. They were performed at traverse speeds that lie entirely above the upper bound of the training range, so no training condition shares their parameter combination or even their region of the parameter space. Their measured feature values enter neither the data-fit loss nor any physics residual, and they take no part in the computation of the standardisation statistics of the targets, which are obtained from the training set alone under the availability mask. No hyperparameter, loss weight or stopping criterion was selected by reference to their performance. They therefore constitute independent experimental validation under previously unseen conditions rather than a partition of the development data. What they do not constitute, and what Section 5 states plainly, is a validation population large or diverse enough to support strong conclusions. Four points varying in a single direction cannot separate competing models, and the leave-one-out cross-validation reported in Section 3.10 is offered as the complementary and more conservative estimate.

#### 2.2.3. Experimental Matrix and Reproducibility

The complete experimental matrix consists of 40 physical experiments (36 training plus 4 pure validation). The parameter levels of the training and pure validation sets are summarised in Table 2.

### 2.3. Image Acquisition and Processing Pipeline

The geometric characterisation of the PBAAM single-track experiments is based on visual data obtained from three different imaging modalities: top-view images of the solidified track (referred to here as E-type), arc images acquired during the process from a side view (A-type) and cross-sectional images of the molten pool taken at the mid-point of the track (M-type). The joint use of these three modalities allows extraction of a richer feature set (top-surface morphology, arc profile and penetration depth) than any single imaging modality could provide; this approach parallels the multi-modality strategies adopted in laser-based single-track studies [28,29].

#### 2.3.1. Image Acquisition Protocols

Top-view images were acquired after each experiment under controlled illumination and constant magnification, after the excess powder had been carefully removed from the powder bed. The camera-to-specimen distance was kept identical across all experiments. These images provide information on track continuity along its length, equivalent track width and surface morphology (sintered region, melted region). Pixel-to-length calibration was performed using the known dimensions of the 10 × 30 mm 316L substrate, which is fully visible in the image frame.

Arc behaviour during the process was recorded with a Sony α7 Mark V digital camera (Sony Corporation, Tokyo, Japan). Owing to the high-intensity radiation of the arc, the camera was fitted with a neutral density (ND) filter and operated at ISO 80, an aperture of f/2.8 and a shutter speed of 1/12,000 s [7]. The camera was positioned in a front-facing configuration parallel to the scanning axis so that the arc profile remained within the field of view throughout each single-track experiment. Pixel-to-length calibration was performed using the tungsten electrode diameter (1.6 mm), which is always visible in the frame.

After top-view imaging had been completed, the mid-region of each track was sectioned in a plane normal to the substrate using a MICRACUT 200-S precision cutting machine (Metkon Instruments Inc., Bursa, Türkiye) to produce cross-sectional specimens. The sectioned specimens were embedded in epoxy resin and then subjected to progressive surface preparation with 800, 1000, 1200 and 2500 grit SiC abrasive papers in sequence. Polishing was performed on a felt pad with diamond suspension, and Vilella’s reagent (5 mL HCl, 1 g picric acid, 100 mL ethanol) was used for etching to reveal the molten-pool boundary. Metallographic observations were carried out on an AOB Optimus optical microscope (AOB TEST Systems/AOBLAB, İstanbul, Türkiye) under a 10× objective using ToupView 4.11 image-analysis software [7].

#### 2.3.2. Otsu Thresholding and Manual Region Delineation

**Figure 2:**
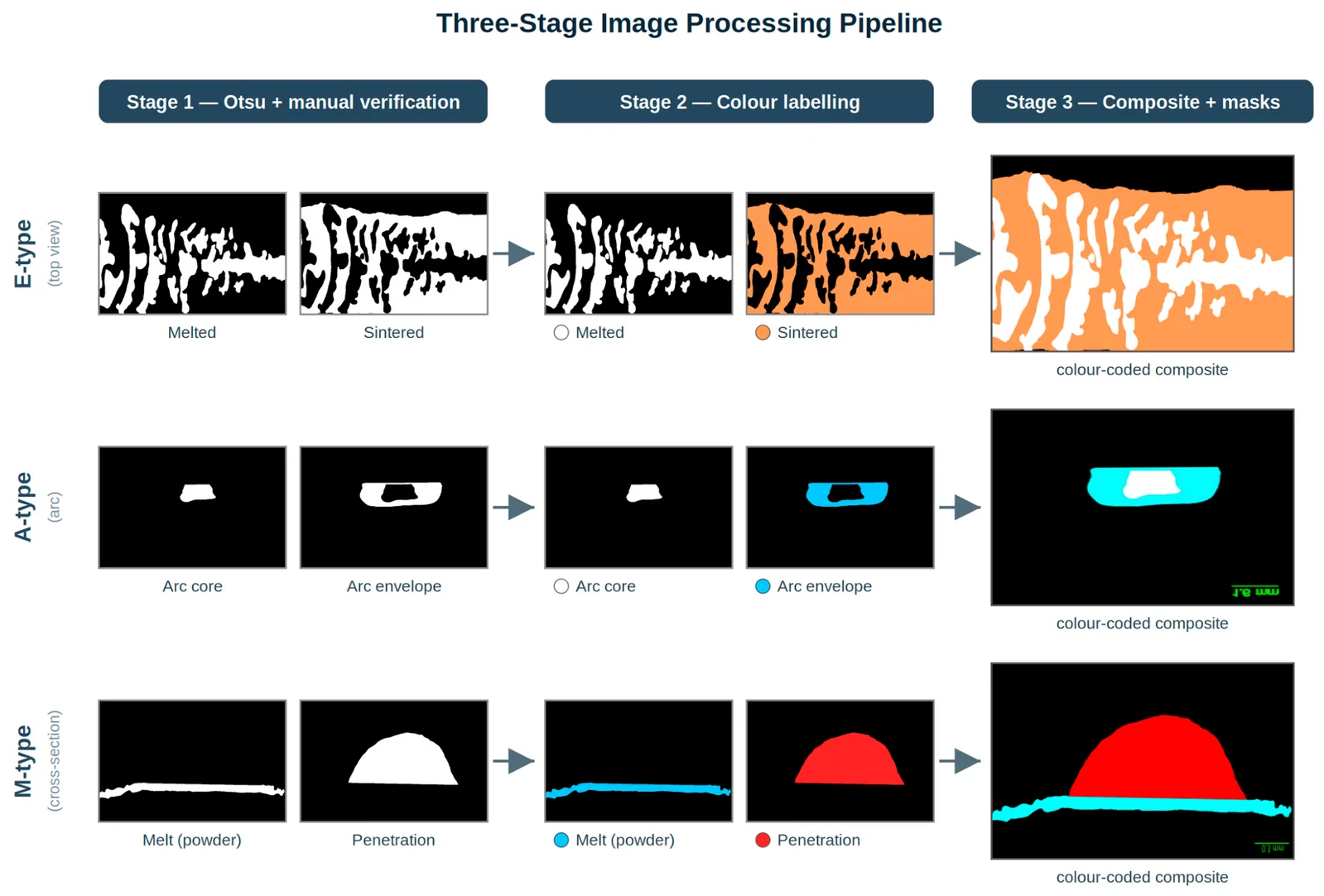
Three-stage image processing pipeline, applied to all three imaging modalities (E-type, A-type, M-type): Stage 1: Otsu global thresholding followed by manual verification, producing a separate binary mask for each semantic region; Stage 2: colour labelling, in which each region is assigned its distinct RGB code; Stage 3: merging of the labelled regions into a single colour-coded composite, from which each semantic class is also retained as an individual binary mask.

The raw images obtained from the three imaging modalities were processed through a three-stage image processing procedure (Figure 2) to separate the semantic regions. This procedure combines classical digital image processing techniques with controlled human supervision, providing robust region identification under unstable track morphologies and low-contrast boundary regions.

In the first stage, the raw images were converted to grayscale and pre-binarised using Otsu’s global thresholding method [30]. Otsu’s method automatically determines the optimum threshold value that minimises the intra-class variance under the assumption of a bimodal histogram and is comparatively robust to illumination variations.

In the second stage, region-based manual delineation was applied to the regions in which Otsu thresholding was found to be insufficient. Such regions, particularly the transition zone where the melted track meets the sintered band, the gradient zone between core and envelope in the arc images, and the etching gradient zones in the cross-sectional images, were observed; for these regions, each semantic class (melted region, heat-affected zone, sintered band, penetration zone and so on) was defined manually as a separate pixel layer. The delineation was performed using the open-source vector-based image-editing software Inkscape (version 1.4, Inkscape Project), with the melt boundary established at the points where it could be unambiguously identified by metallographic inspection.

In the third stage, after each semantic layer had been defined individually, the layers were merged into a single composite image. In this merged image each semantic class is encoded by a distinct RGB colour value (Section 2.3.3). The output is stored both as a single image with a black-and-white background and coloured semantic regions and as a set of individual binary masks for each class.

#### 2.3.3. Colour-Based Region Labelling

**Figure 3:**
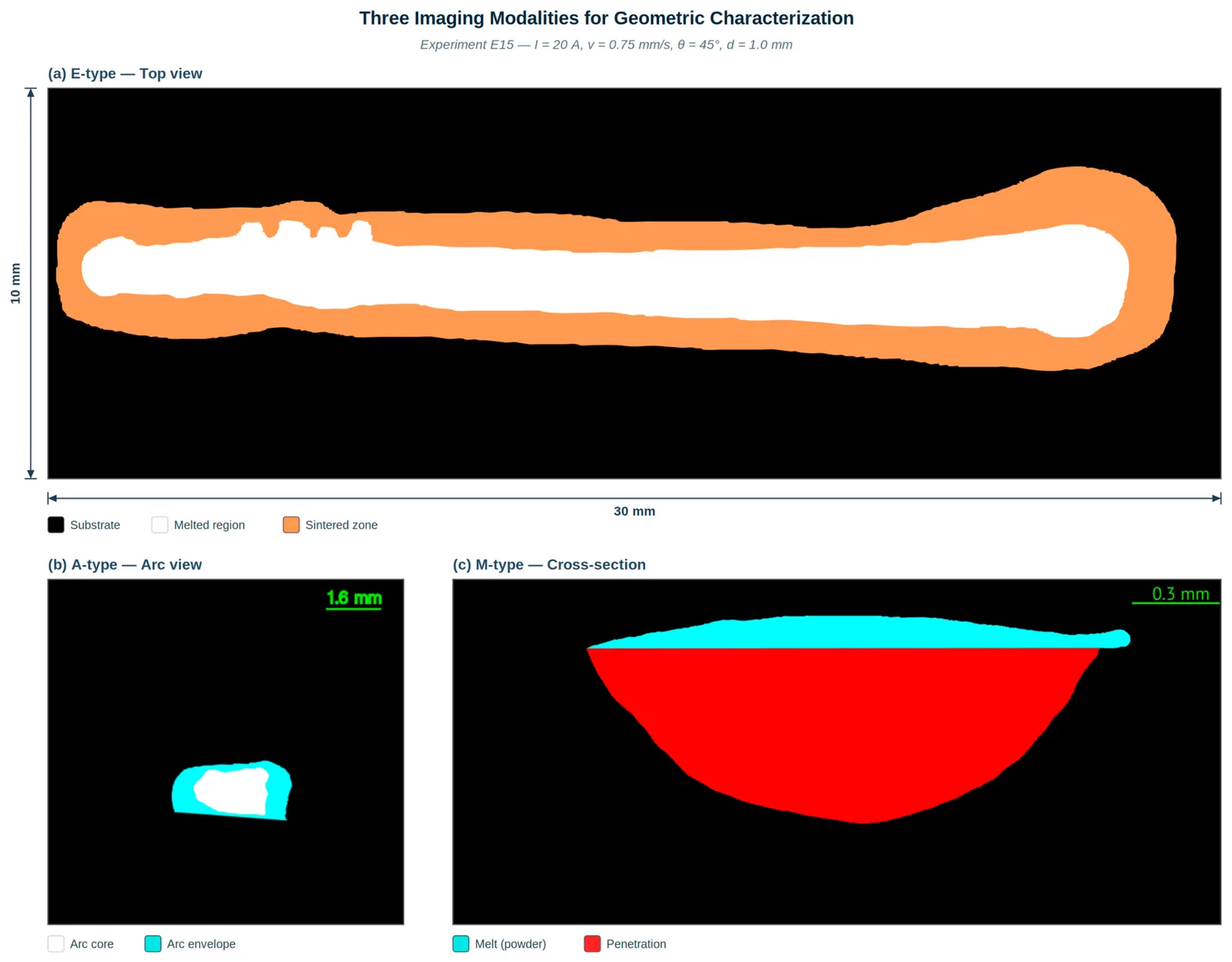
Colour-coded segmentation maps of the three imaging modalities for a representative single-track experiment (E15: I = 20 A, v = 0.75 mm/s, θ = 45°, d = 1.0 mm), each shown at its true aspect ratio: (**a**) top-view (E-type): substrate (black), melted region (white) and sintered region (orange); the frame corresponds to the 10 × 30 mm substrate; (**b**) arc image (A-type): dense arc core (white) and diffuse arc envelope (blue); (**c**) cross-section (M-type): powder-derived solidification (blue), substrate-penetration region (red) and green scale bar (0.3 mm reference).

The semantic layers produced were labelled with distinctive RGB values according to the imaging modality (Figure 3). Three regions were defined in the top-view images: black (substrate), white (the main track that has melted and solidified in the powder bed) and orange (RGB ≈ 255, 153, 85; the region of arc-induced sintering or colour change in the powder). The white region was used in the analysis of equivalent track width (w_iz_) and continuity, while the orange region was used in the derivation of complementary features such as sintered-zone width.

Two regions were defined in the arc images: white (the dense arc core) and blue (the diffuse arc envelope). The union of core and envelope defines the total arc area, while the core area alone defines the high-density core diameter (D_q_) within the arc. Green (RGB ≈ 18, 237, 2) was reserved for the scale bar.

Two principal regions were defined in the cross-section images: blue (RGB ≈ 0, 255, 255; the upper part that has melted and solidified in the powder bed) and red (RGB ≈ 255, 0, 0; the substrate-penetration region). Green (RGB ≈ 3, 255, 3) denotes the scale bar (0.3 mm reference). The union of the blue and red regions defines the total melt-pool cross-section, while the red region alone defines the penetration depth (h_pen_) and penetration area (A_pen_).

#### 2.3.4. Scale Calibration and Reproducibility

The pixel-to-length conversion for the three imaging modalities was derived from reference objects always visible in the image frame: the known dimensions of the 316L substrate (10 × 30 × 2 mm) for the top-view and cross-section images; and the tungsten electrode diameter (1.6 mm) for the arc images. To ensure the reproducibility of the manual delineation step, a single operator processed the regions of boundary uncertainty within the same set of reference rules. To assess operator consistency, an independent re-labelling of a randomly selected subsample from the experimental set was carried out in a second session interval; this re-labelling was compared with the main labelling to verify intra-operator consistency.

### 2.4. Geometric Feature Extraction

From the colour-coded segmentation maps described in Section 2.3, each semantic region was extracted as a separate binary mask, and five geometric features that constitute the output space of the PINN model were derived: arc core diameter (D_q_), arc cone angle (α_c_), heat-affected zone width (w_HAZ_), track core width (d_core_) and equivalent track width (w_iz_). The first two features are extracted from the arc image, the last three from the top-view image; the penetration features (h_pen_, A_pen_) obtained from the cross-section images were not used as PINN model outputs in this study but are reported for complementary verification purposes.

#### 2.4.1. Features Extracted from the Arc Image

In the arc images the white core region represents the high-density plasma column, while the blue envelope region represents the diffuse arc-flux distribution. The arc core diameter (D_q_) is defined as the length of the widest horizontal section of the white core region taken along the electrode axis. This quantity reflects the maximum constriction diameter of the arc column and is used in the literature as an indicator of current density in TIG arcs [22]. Because the cone angle defined below is positive throughout the dataset, the core widens from the electrode tip toward the bed; the widest section, from which the core diameter is measured, therefore lies near the bed surface and exceeds the constriction diameter near the electrode tip quoted in Section 2.1.1.

The arc cone angle (α_c_) is defined as the apex angle of the profile of the white core region as it widens from the electrode tip toward the powder-bed surface. This angle is computed using the geometric ratio of the widths measured at the upper half (electrode side) and the lower half (bed side) of the core region:

*α_c_* = 2 · *arctan*[(*w_bottom_* − *w_top_*)/(2 · *h_arc_*)] (1)

Here *w*_top_ and *w*_bottom_ are the widths at the upper and lower bounds of the core region, respectively, while *h*_arc_ is the vertical distance between the two bounds.

The heat-affected zone width (*w*_HAZ_) is measured on the top-view image and is defined in Section 2.4.2. The arc envelope, that is the blue region surrounding the core, was segmented as well but yields no model output; it serves only to delimit the core region for the two features defined above.

#### 2.4.2. Features Extracted from the Top-View Image

The white region of the top-view images represents the main melt track that has melted and solidified in the powder bed. To compute the local width profile, each column (cross-section perpendicular to the X-axis) of the binary mask was scanned individually; the vertical distance between the Y coordinates of the topmost and the bottommost white pixels in each column was recorded as the local track height *w*(*x*) at that point.

The track core width (*d*_core_) is defined as the arithmetic mean of the local width profile along the build direction:

*d_core_* = (1/*N_x_*) · *Σ w*(*x_i_*) (2)

**Figure 4:**
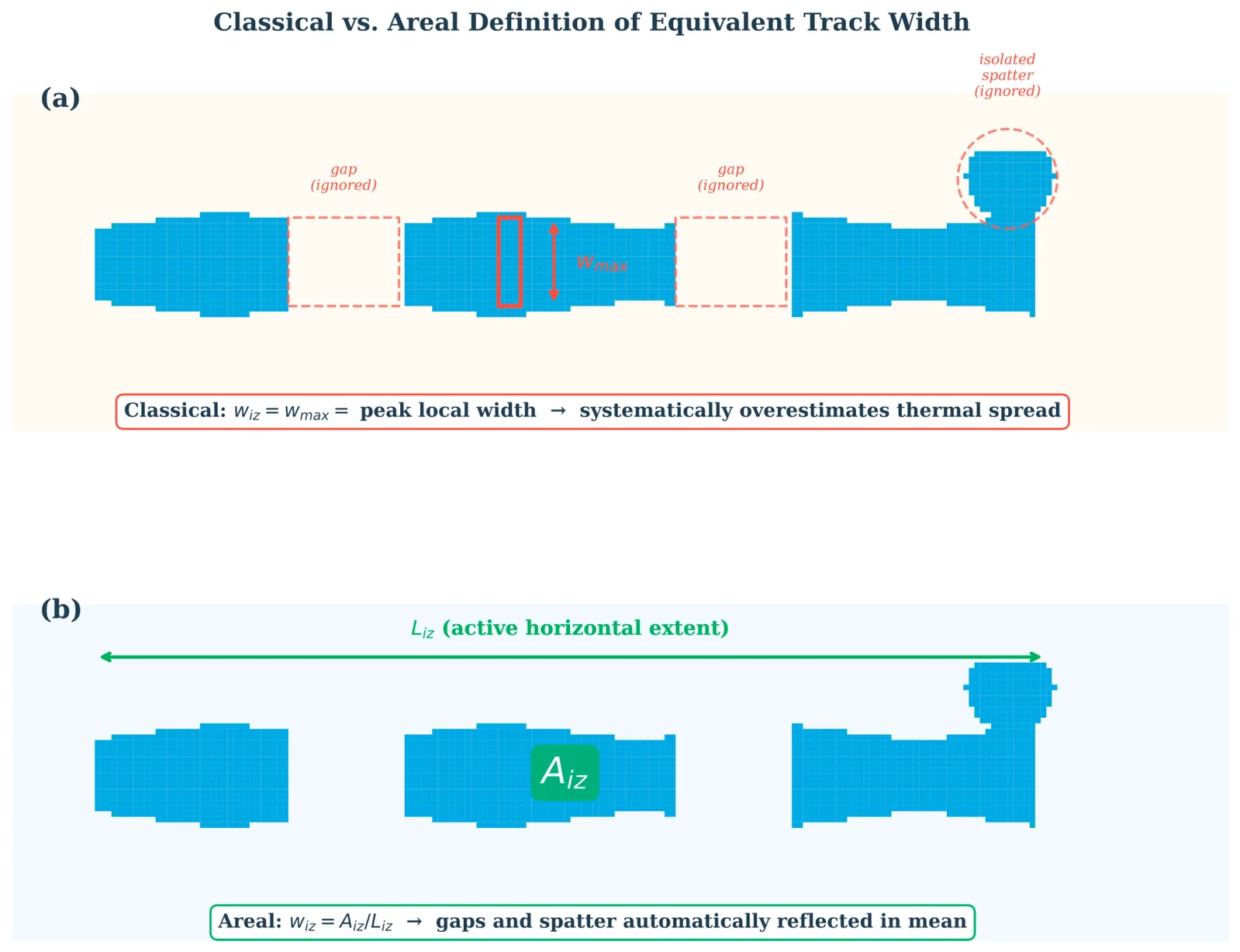
Comparative schematic of the classical maximum-width approach with the areal definition of the equivalent track width (w_iz_ = A_iz_/L_iz_) proposed in this study: (**a**) on a track example containing a gap, the classical maximum-width definition overestimates the thermal spread of the track by ignoring local constrictions; (**b**) the areal definition automatically reflects both gaps and agglomerations in the mean, providing a true geometric representation of the track.

The equivalent track width (*w*_iz_) is an areal definition proposed in this study. In the classical approach, track width is taken as the maximum horizontal extent of the track; however, this definition does not reflect track gaps, agglomerations and local constrictions. Under the unstable track morphologies frequently observed in PBAAM processes, the maximum-width approach tends to systematically overestimate the actual thermal spread of the track (Figure 4). To prevent this systematic bias, the equivalent track width is defined as:

*w_iz_* = *A_iz_*/*L_iz_* (3)

Here, *A*_iz_ is the total area of the white region in the top-view image (mm^2^) and *L*_iz_ is the active horizontal extent of the track along the build direction (the distance between the first and the last column containing the main track, mm). By definition, *w*_iz_ is the areal mean of the track, so that gaps and agglomerations are automatically reflected in the mean [8].

The heat-affected zone width (w_HAZ_) is measured on the same top-view image as the outer boundary of the combined white and orange regions, that is of the melted track together with the surrounding band of sintered or discoloured powder. It is obtained as the mean of the column-wise extent of that combined region over the active track length, in the same manner as the local width profile above. This feature measures the thermal footprint of the arc on the powder bed and therefore indicates how much loose powder is bonded at the periphery of the deposit. By construction, it is not smaller than the width of the melted track alone, which is the ordering imposed by the first geometric constraint in Section 2.7.3.

#### 2.4.3. Morphological Stability Metrics

**Figure 5:**
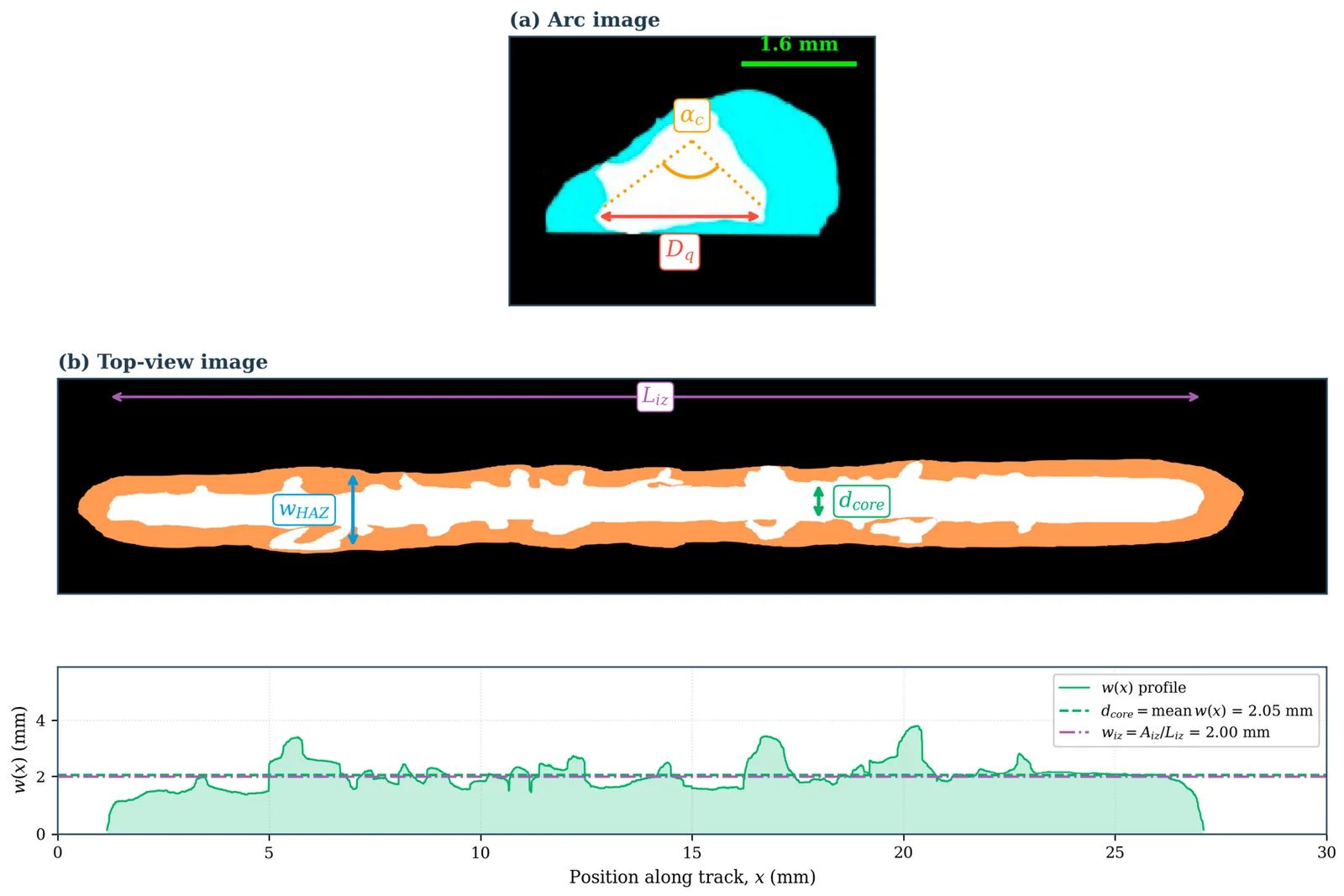
Visual illustration of the five geometric features defined in the PBAAM single-track experiments: (**a**) on the arc image: D_q_ (arc core diameter), α_c_ (arc cone angle); (**b**) on the top-view image: w_HAZ_ (heat-affected zone width), d_core_ (track core width), w_iz_ (equivalent track width) and the local width profile w(x) from which they are derived.

The five geometric features defined above are illustrated together in Figure 5. In addition to these, four complementary metrics quantifying the morphological stability of the track were derived. These metrics are not used directly as PINN model outputs but constitute the quantitative inputs of the quality filter algorithm defined in Section 2.5.

The continuity metric (C) is defined as the ratio of columns containing white pixels along the active track extent: C = L_iz,white_/L_iz_. The condition C = 1 indicates that the track is fully continuous, while C < 1 indicates the presence of horizontal discontinuities (gaps) along the main track [7].

The width coefficient of variation (CV) is computed as the ratio of the standard deviation to the mean of the local width profile: CV = σ_w_/μ_w_. This metric is a dimensionless measure of the geometric stability of continuous tracks [7,12].

The agglomeration count (N_a_) is defined as the number of independent white regions outside the main track, identified by connected component labelling on the binary mask.

The linearity-deviation metric (σ_cl_) measures the deviation of the central axis of the track from a straight line. The mid-Y coordinate y_c_(x) = (y_min_ + y_max_)/2 of the track was computed for each column; a linear fit was applied to this point sequence, and the standard deviation of the deviations of the observed y_c_(x) values from this fit was taken as σ_cl_.

### 2.5. Quality Filter: Stable/Unstable Classification

Two principal modes of morphological instability are observed in the PBAAM single-track experiments, paralleling classical single-track studies, namely gap formation and excessive width fluctuation (balling). These instabilities have different origins in melt-pool physics, but their common consequence is to drive the geometric quantities of the track (in particular the equivalent track width w_iz_) away from a physics-consistent relation with the process parameters.

#### 2.5.1. Physical Basis of the Morphological Metrics

The four morphological metrics defined in Section 2.4.3 correspond to four different instability modes recognised in arc-based melt-pool physics. Continuity (C) relates to the longitudinal integrity of the track; gap formation occurs under conditions where the arc-flux density is insufficient or the solidification time of the melt pool is unbalanced with the scan speed [8].

The width coefficient of variation (CV) is a quantitative indicator of the Plateau–Rayleigh instability [25]. The Plateau–Rayleigh instability is the tendency of the melt pool to break up into independent droplets under the influence of surface tension; it becomes dominant when the ratio of melt-pool length to width exceeds a certain critical value, leading to the periodic narrowing–broadening fluctuations known as balling [12,31].

The agglomeration count (N_a_) is associated with the solidification of droplets ejected from the melt pool under unstable arc conditions as independent spherical powder-bed agglomerates (spatter) outside the main track [32]. The linearity deviation (σ_cl_) reflects the lateral drift (waviness) of the track from the build direction caused by instabilities of the arc column or electrode positioning [10].

#### 2.5.2. Composite Quality Score and Classification

The four morphological metrics were transformed into sub-scores scaled to the [0, 1] interval and combined in a weighted composite quality score S:

*s*_1_ = *C*,   *s*_2_ = 1/(1 + 5·*CV*),   *s*_3_ = 1/(1 + 0.3·*N_a_*),   *s*_4_ = 1/(1 + 5·*σ_cl_*) (4)

The composite quality score *S* is defined as the weighted linear combination of the sub-scores:

*S* = 0.30·*s*_1_ + 0.30·*s*_2_ + 0.20·*s*_3_ + 0.20·*s*_4_ (5)

The continuity (*s*_1_) and width-fluctuation (*s*_2_) weights are higher (0.30 each) because of the direct physics-based effect of these two metrics on the equivalent track width; the agglomeration count (*s*_3_) and linearity deviation (*s*_4_) weights are lower (0.20 each) because these two metrics play a complementary role.

**Figure 6:**
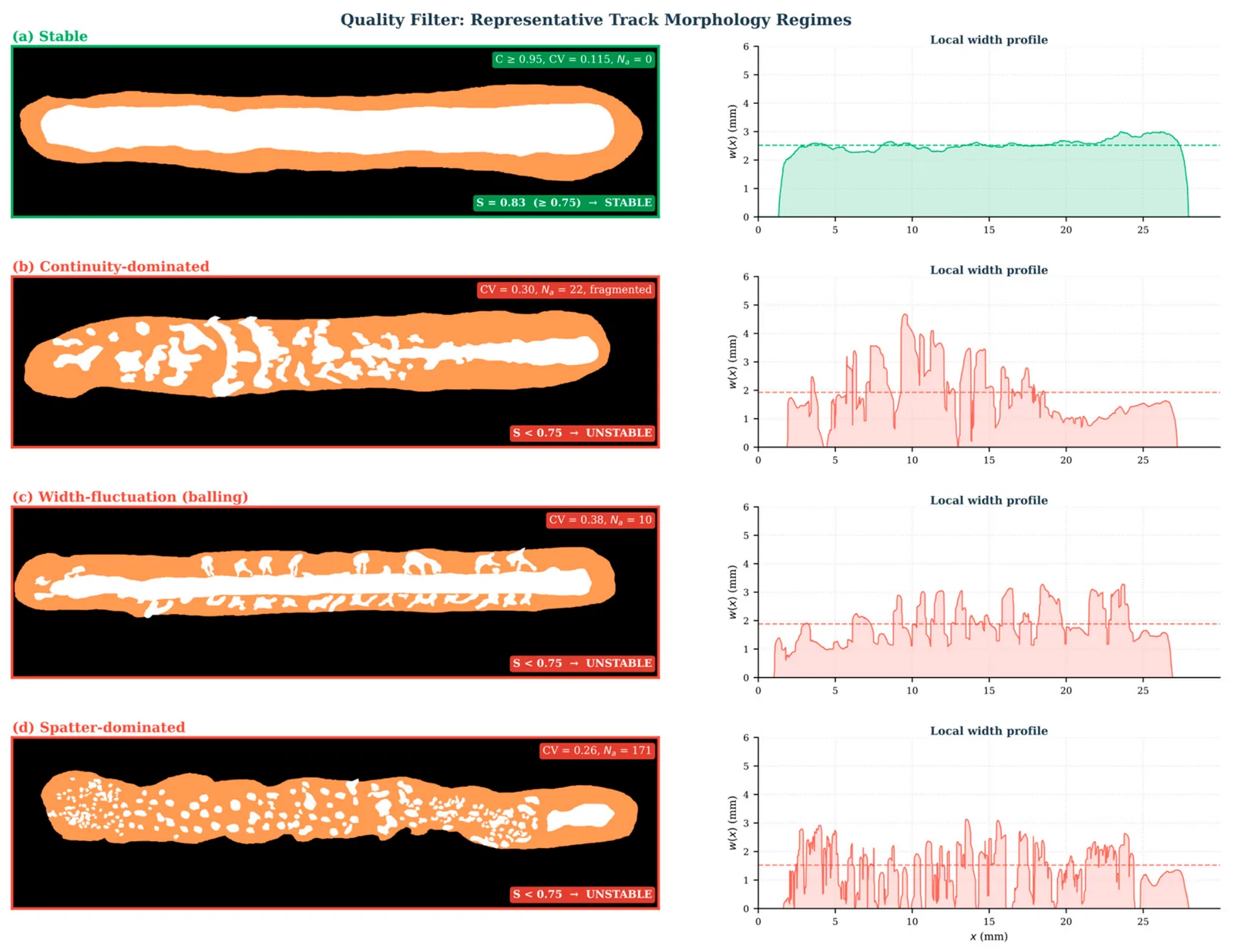
Representative example images and corresponding local width profiles *w*(*x*) for the track morphology regimes distinguished by the quality filter: (**a**) stable track: composite quality score *S* ≥ 0.75; continuous, uniform-width and agglomeration-free morphology; (**b**) continuity-dominated unstable track: fragmented solidification with composite score below the threshold due to low continuity; (**c**) width-fluctuation (balling) dominated unstable track: periodic narrowing–broadening fluctuation, filtered out due to the high coefficient of variation; (**d**) spatter-/agglomeration-dominated track: filtered out by high agglomeration count (*N*_a_) and linearity deviation (*σ*_cl_). In the width profiles, the horizontal dashed line marks the mean track width; green shading denotes the stable track and red shading the unstable tracks.

Track classification is performed by a binary threshold decision on the composite score. Tracks satisfying *S* ≥ 0.75 are labelled stable, while tracks not satisfying it are labelled unstable (Figure 6). The threshold value of 0.75 corresponds to a conservative quality criterion: if the four sub-scores are equal it requires each to reach 0.75, and a pronounced deficit in any single sub-score can be compensated only by near-perfect values of the remaining three.

#### 2.5.3. Role of the Filter in PINN Training

The direct effect of the quality filter is that the w_iz_ output is updated during PINN model training only with data from stable tracks. The other four geometric features (D_q_, α_c_, w_HAZ_, d_core_) can be measured consistently in all 36 training experiments independently of the stability conditions, so their training loss is computed over the entire dataset. By contrast, w_iz_ is included in the training loss only on the samples that satisfy the filter conditions because it falls in area on unstable tracks and produces inconsistencies with the physical constraints. Of the 23 tracks classified as stable, the areal width enters the loss for 21; for the remaining two the areal width could not be evaluated reliably, and the corresponding entries are excluded by the availability mask described in Section 2.6.5.

This selective training approach falls into the category of quality-aware loss configuration that has been described in the physics-informed learning literature; it prevents noisy or out-of-regime data points from corrupting the training signal of the physics constraint [21].

### 2.6. PINN Architecture

**Figure 7:**
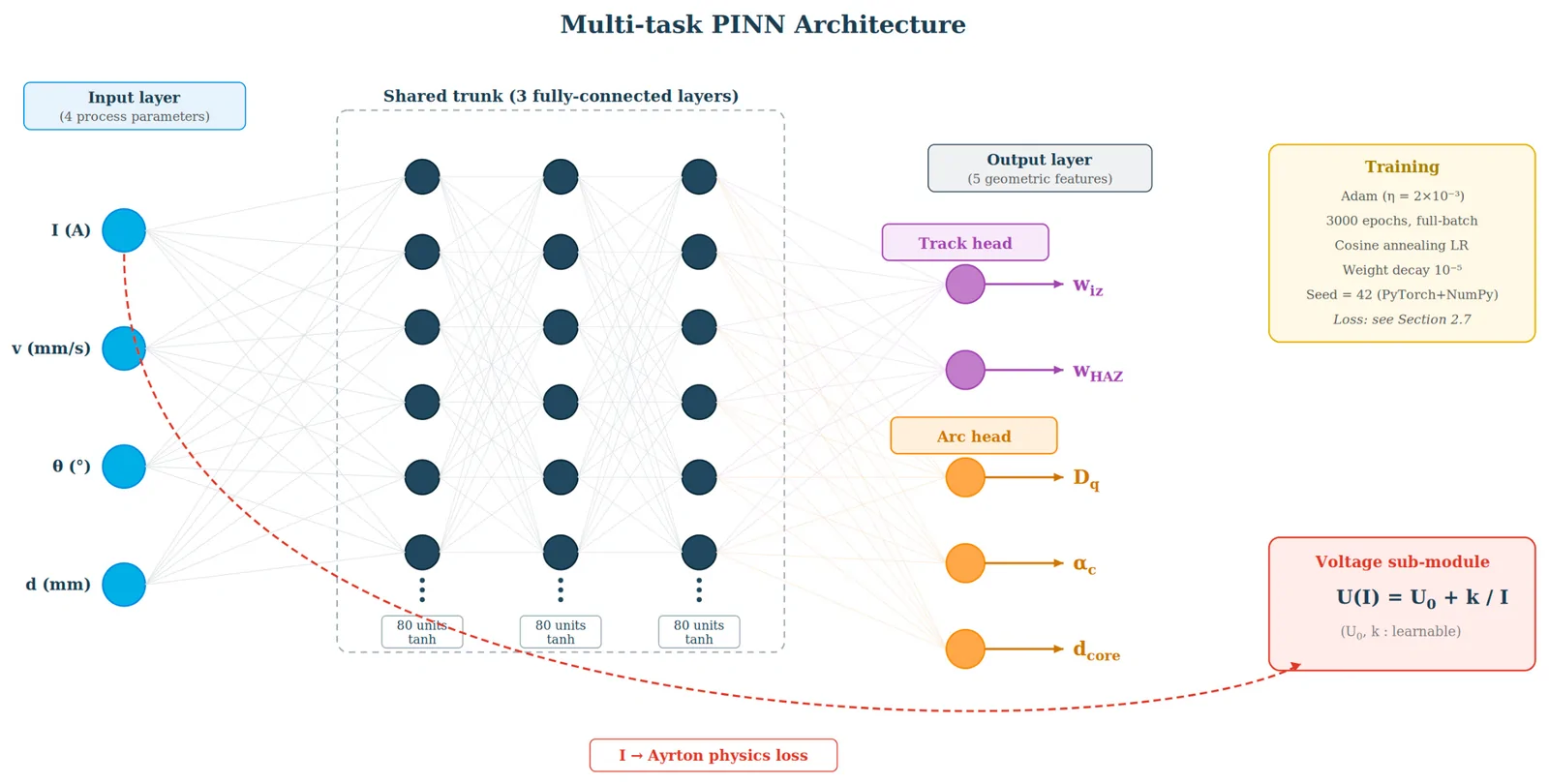
Schematic of the proposed multi-task PINN architecture. The input layer contains the four process parameters (I, v, θ, d); the shared trunk contains three sequential fully connected layers of 80 neurons each (with tanh activations); the two separate task heads contain the top-view width features (w_iz_, w_HAZ_) and the remaining features (D_q_, α_c_, d_core_). The voltage sub-module (U_0_, k) is integrated into the network as a separate learnable component for the Ayrton physics loss.

A multi-task Physics-Informed Neural Network (PINN) was designed that can simultaneously predict five geometric features from the four process parameters. The architecture is built on a shared-trunk approach in which two distinct task heads diverge from a shared latent representation (Figure 7). This approach offers superior performance, in terms of both data efficiency and physical consistency, over independent single-output models in applications where the parameter space is small and the physical constraints are consistent across outputs [12,16].

#### 2.6.1. Network Topology

The network has a shared trunk consisting of three sequential fully connected hidden layers of 80 neurons each, beginning with a 4-neuron input. A hyperbolic tangent (tanh) activation function is used in each hidden layer. The tanh activation is preferred for two main reasons: first, in contrast to piece-wise-linear activations such as ReLU, it is infinitely differentiable everywhere, providing the mathematical consistency required when the physics loss terms involve derivatives of the network output [14]; second, its symmetric output centred on the [−1, 1] range supports balanced gradient flow on small datasets [15].

In the terminology of multi-modal learning, this is an intermediate, feature-level fusion architecture. Early fusion, in which raw modality data are concatenated before any learning, is not applicable here because the network input is the four process parameters rather than the images themselves; the imaging modalities enter on the output side, as the sources from which the five targets are measured. Late fusion, in which a separate model is fitted per output and the predictions combined afterwards, was rejected because it cannot express the ordering relations that couple features measured on different images, and the ablation reported in Section 3.8 confirms that this coupling is where most of the benefit lies. The intermediate scheme adopted here shares a single latent representation across all five targets and specialises only in the final linear layer, which allows the targets to regularise one another under a small sample while leaving each output its own scale. The output of the shared trunk is routed to two task heads. It should be noted that the grouping of the heads is by feature family rather than strictly by imaging modality: the first head collects the two width features measured on the top-view image, while the second collects the two arc-image features together with the track core width, which is also measured on the top-view image. The first head, a binary (2-output) linear layer producing the two width features measured on the top-view image, predicts the equivalent track width (w_iz_) and the heat-affected zone width (w_HAZ_). The second head, a ternary (3-output) linear layer, predicts the arc core diameter (D_q_), the arc cone angle (α_c_) and the track core width (d_core_) [17].

The voltage–current relation, required for the Ayrton physics loss detailed in Section 2.7, is integrated into the network as a separate learnable sub-module. This sub-module contains two learnable parameters (U_0_ and k) following the classical Ayrton equation, and is trained jointly with the other parameters of the network.

#### 2.6.2. Training Strategy

The network weights were optimised with the Adam (Adaptive Moment Estimation) algorithm [33]. The training parameters were set to an initial learning rate of 2 × 10^−3^, weight decay of 1 × 10^−5^ and a total training duration of 3000 epochs. The weights of all linear layers were initialised with the PyTorch 2.13.0 default scheme for fully connected layers, that is a Kaiming uniform distribution, and the two parameters of the embedded voltage sub-model were initialised deterministically at U_0_ = 14.0 V and k = 100.0 V·A from the order of magnitude expected for a low-current arc. The model contains 13,767 trainable parameters in total, of which 13,765 belong to the network and 2 to the voltage sub-model, giving 382 parameters per training sample; the consequences of this ratio are discussed in Section 5. The learning rate, the number of epochs, the network depth and width and the four loss weights were selected empirically, by trial and adjustment against validation performance, and not by a systematic grid or random search; the sensitivity analysis of Section 3.9 was carried out after the fact and confirms that every perturbation of an active loss weight degrades performance, so the nominal values sit at a local optimum. The batch size was not a free choice: with 36 training conditions, the full set is presented in a single batch at every step and no mini-batching is used. Weight decay was applied to the network parameters only and explicitly excluded from the two physical parameters, so that their displacement during training originates from the physics loss rather than from regularisation. Throughout training, the learning rate was reduced progressively according to a cosine-annealing schedule [34].

To ensure reproducibility of the training and inference processes, the random seed values of PyTorch and NumPy were fixed to a single value (42).

#### 2.6.3. Training and Validation Data Split

Network training was performed on the 36 training experiments defined in Section 2.2; the four pure validation experiments were kept entirely separate from the training process. A split into train/validation/test subsets within the training set, common in classical machine learning approaches, was not employed in this study. Instead, the validation task was performed exclusively by out-of-distribution points.

#### 2.6.4. Software Implementation of the Network Training

All network design, training pipeline and physics-loss implementations were developed in the PyTorch 2.13.0 deep-learning framework [35]. The torch.optim.Adam module of PyTorch was used for Adam optimisation, while the torch.optim.lr_scheduler.CosineAnnealingLR module was used for the cosine-annealing learning-rate schedule. The gradient computations required by the physics-loss terms were managed via PyTorch’s automatic differentiation (autograd) mechanism. Training was carried out on the CPU; GPU acceleration was not necessary owing to the small size of the training set (36 samples) and the absence of mini-batch partitioning.

#### 2.6.5. Preprocessing and Reproducibility

The four process parameters were scaled to the interval from minus one to plus one by a min-max transformation whose bounds were taken over the input parameters of the training and validation conditions jointly rather than over the training conditions alone, so that the extrapolated traverse speeds map inside the interval instead of beyond it. This choice concerns the input scaling only. No target information from the validation set enters the transformation, and the standardisation statistics of the targets are computed over the training set alone, as described below. The validation targets therefore play no part in fitting the model. We state the choice explicitly because scaling bounds that depend on the validation inputs are a mild departure from the strict convention of deriving every preprocessing constant from the training set alone. The five targets were standardised individually by subtracting the mean and dividing by the standard deviation, both computed over the training set only and both computed under the availability mask, so that conditions for which a given target is absent contribute neither to the mean nor to the standard deviation of that target. Missing targets are handled by the mask rather than by imputation. The areal track width is absent for the unstable tracks, and the corresponding entries are excluded from the data-fit loss and from the heat-conduction residual by multiplication with a binary mask, so that no surrogate value is ever fitted. Predictions are clipped at zero on inversion of the standardisation, since all five features are non-negative by definition. Noise removal is performed at the image stage and not on the extracted features. The morphological operations, the thresholding and the removal of isolated components are described in Section 2.3, and no smoothing or outlier rejection is applied to the feature values themselves.

The implementation uses PyTorch for the model and the optimiser, NumPy for the numerical operations, pandas for data handling and scikit-learn 1.9.0 for the input scaler. Random number generators of PyTorch and NumPy were seeded at 42, and the training uses full-batch gradient descent, so a run repeats exactly on the same platform. The dataset comprising the four process parameters and the five extracted features for all 40 experiments, together with the training script, will be made available by the corresponding author on reasonable request.

### 2.7. Physics-Based Loss Function

Before the individual terms are defined, the nature and the scope of the physical information they carry should be stated. The constraints used here are not the residual of a governing partial differential equation. They are of three kinds. The first is a scaling relation derived from the classical theory of a moving heat source, which constrains how the melted width must respond to the energy delivered per unit length. The second kind consists of geometric ordering relations that follow from the definitions of the measured features and must hold for any physically realisable track. The third kind consists of admissibility bounds and sign conditions on quantities whose physical range is known independently of the present measurements. No fluid-flow or mass-conservation term is included. The melt pool in this process is small and short-lived, its internal convection was not instrumented, and a Marangoni or buoyancy term could not be validated against anything measured here. Adding an unvalidated flow term would introduce parameters that the 36 training conditions cannot identify. The constraints retained are therefore those that are either derivable from first principles at the level of energy balance, or entailed by the geometry of the measurement itself, and the sensitivity analysis in Section 3.9 quantifies how much each contributes. The residuals themselves are summarised in Table 3, and the material and thermophysical property values that enter them are collected in Table 4.

The thermophysical properties are treated as temperature-independent. This is a deliberate simplification and its justification is that the residual in which they appear is a scaling relation rather than a field solution. The properties enter only through the product ρ_eff_H_m_, which sets the energy required to melt unit volume, and the residual is in any case evaluated against a proportionality constant that is refitted during training. Temperature-dependent property data would refine the absolute value of that product but would be absorbed into the fitted constant and would therefore not change the constraint imposed on the network. The powder particle size distribution and the chemical composition of the feedstock are given in Section 2.1.2.

No boundary conditions in the sense of a field problem arise, since no field is solved. The heat-conduction residual is a scaling relation evaluated pointwise at the 36 training conditions, and the constant of proportionality *k*_w_ relating the predicted width to the scaling variable is refitted at every optimisation step rather than fixed a priori; the consequence of that choice for parameter identifiability is discussed in Section 4.3.

The total loss function used in PINN model training is defined as the weighted linear combination of five components: the data fit loss (*L*_data_), the Rykalin-type heat-conduction scaling loss (*L*_phys_), the geometric consistency loss (*L*_geom_), the monotonicity loss (*L*_mono_) and the voltage box-constraint loss (*L*_volt_):

*L* = *L_data_* + *λ_phys_*·*L_phys_* + *λ_geom_*·*L_geom_* + *λ_mono_*·*L_mono_* + *λ_volt_*·*L_volt_* (6)

This structure follows the multi-component loss formulation common in the physics-informed neural network literature [14,15]; however, in that the physics constraint is formulated not as a single partial differential equation but over four different physical dimensions of the melt-pool geometry, it represents an extended application of the loss function modification category of [21].

#### 2.7.1. Data Fit Loss

The data fit loss *L*_data_ expresses the standardised mean squared error between the network predictions and the experimental measurements:

*L_data_* = *Σ M_ij_* · (*ŷ_ij_* − *y_ij_*)^2^/*Σ M_ij_* (7)

Here, *ŷ*_ij_ is the network prediction; *y*_ij_ is the experimental value; and *M*_ij_ is a binary mask that takes the value zero for missing or filtered observations and one for valid observations. Unstable tracks are masked with *M* = 0 in the *w*_iz_ target column.

#### 2.7.2. Rykalin-Type Heat-Conduction Scaling Loss

The physically consistent scaling of the track width with the heat input is formulated as a constraint based on a Rykalin-type analytical solution. When the temperature field generated by a moving point heat source on a semi-infinite medium is taken to be at the melting isotherm at the boundary, the dependence of the lateral spread of the track on the energy input and the speed scales as:

*w_phys_ ∝* √(*Q_eff_*/(*ρ_eff_* · *H_m_* · *v*)) (8)

The effective energy *Q*_eff_ is defined as the projection of the arc power along the work-angle direction with the working-distance correction:

*Q_eff_* = *η* · *U*(*I*) · *I* · *cos*(90° − *θ*)/(1 + 0.15·*d*) (9)

Here *η* = 0.75 is adopted as a representative arc efficiency for TIG; because the proportionality constant of the scaling relation is refitted at every optimisation step (Equation (11)), the absolute value of η is absorbed into it and does not affect the constraint. *U*(I) is the output of the voltage sub-module that is learned simultaneously by the network. The volumetric melting enthalpy *H*_m_ is computed as the total energy required to heat 316L stainless steel from room temperature to the liquidus and to fuse it:

*H_m_* = *c_p_* · (*T_L_* − *T*_0_) + *L_f_* (10)

The values *c*_p_ = 500 J/(kg·K), *T*_L_ = 1708 K, *T*_0_ = 298 K and *L*_f_ = 260 kJ/kg are the literature values for 316L [36]. The effective density of the powder bed is taken as *ρ*_eff_ = 4235 kg/m^3^, which corresponds to about 53% of the bulk density of 316L [26].

The agreement between the track width and the physical scaling is expressed via a scaling coupling constant *k*_w_:

(11) $$L_{phys}=\Sigma \cdot M_{iz}\cdot {\left({\hat{w}}_{iz}-k_{w}\cdot {\tilde{w}}_{phys}\right)}^{2}/\Sigma \cdot M_{iz}$$

#### 2.7.3. Geometric Consistency Loss

Three physical ordering relations among the five geometric features were added to the loss function as soft inequality constraints: *L*_geom_ = *g*_1_ + *g*_2_ + 0.5·*g*_3_. The first constraint (*g*_1_) imposes the requirement that the heat-affected zone be wider than the melted track [11]. The second constraint (*g*_2_) imposes the requirement that the arc core diameter be larger than the track core width. The third constraint (*g*_3_) expresses the ratio between the track core width and the equivalent track width.

#### 2.7.4. Monotonicity Loss

The physical intuition that the track core width and the equivalent track width should not decrease as the arc current increases was applied as a one-sided constraint on the partial derivatives of the model outputs with respect to the current:

(12) $$L_{\mathrm{mono}}=E[\mathrm{ReLU}(-\partial {ŵ}_{\mathrm{iz}}/\partial I{)}^{2}]+E[\mathrm{ReLU}(-\partial {\hat{d}}_{\mathrm{core}}/\partial I{)}^{2}]$$

Here, the partial derivatives are computed via PyTorch’s automatic differentiation (autograd) mechanism.

#### 2.7.5. Ayrton Voltage Model and Box-Constraint Loss

The current–voltage relation in TIG arcs has historically been represented by the Ayrton equation. This negative differential resistance behaviour, summarised as the decrease in arc voltage with increasing current, is associated in low-current TIG arcs with the steep rise in the near-cathode voltage drop and the contraction of the arc column as the current decreases [22]:

*U*(*I*) = *U*_0_ + *k*/*I* (13)

Here, *U*_0_ and *k* are two scalar parameters that are learned simultaneously with the PINN weights during training; their initial values were set to *U*_0_ = 14 V and *k* = 100 V·A, respectively. To keep the voltage sub-module within a physically reasonable range, a box constraint on *U*(*I*) (8 ≤ *U* ≤ 30 V) and a one-sided positivity constraint on the parameter k were applied.

#### 2.7.6. Lambda Weights

For the balance weights (λ values) of the five loss components, the values *λ*_phys_ = 0.20, *λ*_geom_ = 0.25, *λ*_mono_ = 0.10 and *λ*_volt_ = 0.05 were adopted. This ordering reflects that geometric consistency dominates the training signal, while heat-conduction scaling is held at a moderate weight and the lower-impact monotonicity and voltage box-constraint losses play a supporting role.

### 2.8. Statistical Validation: Bootstrap Confidence Intervals

Because the performance of the PINN model on the pure validation set is evaluated on only four out-of-training-range examples, it is methodologically necessary to quantify the sample-size-dependent variance of the point estimates (mean error values). To this end, the 95% confidence intervals (CIs) of the validation performance were computed using the non-parametric and distribution-free resampling procedure known as bootstrap [37,38].

#### 2.8.1. Definition of Error Metrics

The validation performance is reported through two complementary error metrics for each geometric feature. The primary metric, the Mean Absolute Percentage Error (MAPE), is defined as a scale-independent indicator of relative error:

*MAPE* = (100/*n*) · *Σ*|*ŷ_i_* − *y_i_*|/*max*(*y_i_*, *ε*) (14)

The secondary metric, the Mean Absolute Error (MAE), expresses the absolute error magnitude of the prediction in its own physical unit:

*MAE* = (1/*n*) · *Σ* |*ŷ_i_* − *y_i_*| (15)

#### 2.8.2. Bootstrap Resampling Procedure

Considering the limited sample size in the validation set, the sampling variance of the point estimates of the error metrics was quantified by the bootstrap with replacement. For each geometric feature j, B = 1000 independent bootstrap resamples were drawn from the n_j_ samples in the validation set.

The 95% confidence intervals were computed by the percentile method on the bootstrap distribution:

*CI*_95_% = [*Q*_2.5_%(θ*), *Q*_97.5_%(θ*)] (16)

The percentile method was preferred because it does not require any distributional symmetry assumption and provides a robust confidence-interval estimate for small sample sizes [38]. To ensure the reproducibility of the bootstrap sampling, the NumPy random generator seed was fixed to a single value (42).

#### 2.8.3. Stable–Unstable Comparison on the Training Data

In addition to the validation set, the bootstrap procedure was also applied to compare the distributions of the equivalent track width (w_iz_) of the stable and unstable subsets within the training set. This complementary evaluation provides a quantitative verification that the composite quality score classification defined in Section 2.5 functions as a discriminating filter on the numerical distributions of the geometric features.

#### 2.8.4. Software Implementation of the Statistical Analysis

All statistical computations were performed in a custom computational pipeline on Python 3.14.2 using the NumPy 2.5.1 [39] and Pandas 3.0.5 [40] libraries. Bootstrap distributions and percentile estimation were applied via the numpy.percentile and numpy.random.default_rng.choice routines.

## 3. Results

This section reports the performance of the multi-task PINN model on the PBAAM single-track process, in line with the methodology defined in Section 2. The results are presented under eight subsections: the effect of process parameters on track morphology, results of the composite quality score classification, morphological analysis of the pure validation set, the prediction accuracy of the PINN model, the speed extrapolation curve, the learned Ayrton voltage sub-module, geometric consistency among outputs, and limitations of the method.

### 3.1. Effect of Process Parameters on Single-Track Morphology

In the PBAAM single-track experiments, the effects of the four independent process parameters (arc current I, traverse speed v, work angle θ and working distance d) on track morphology were systematically observed. Evaluated through the local width profile w(x) and the areal equivalent track width w_iz_ metrics defined in Section 2.4, the training set experiments showed distributions in the ranges w_iz_ = 1.18–3.46 mm, w_HAZ_ = 2.93–6.19 mm, D_q_ = 2.10–8.25 mm, d_core_ = 1.88–3.29 mm and α_c_ = 29.95–52.31°.

**Figure 8:**
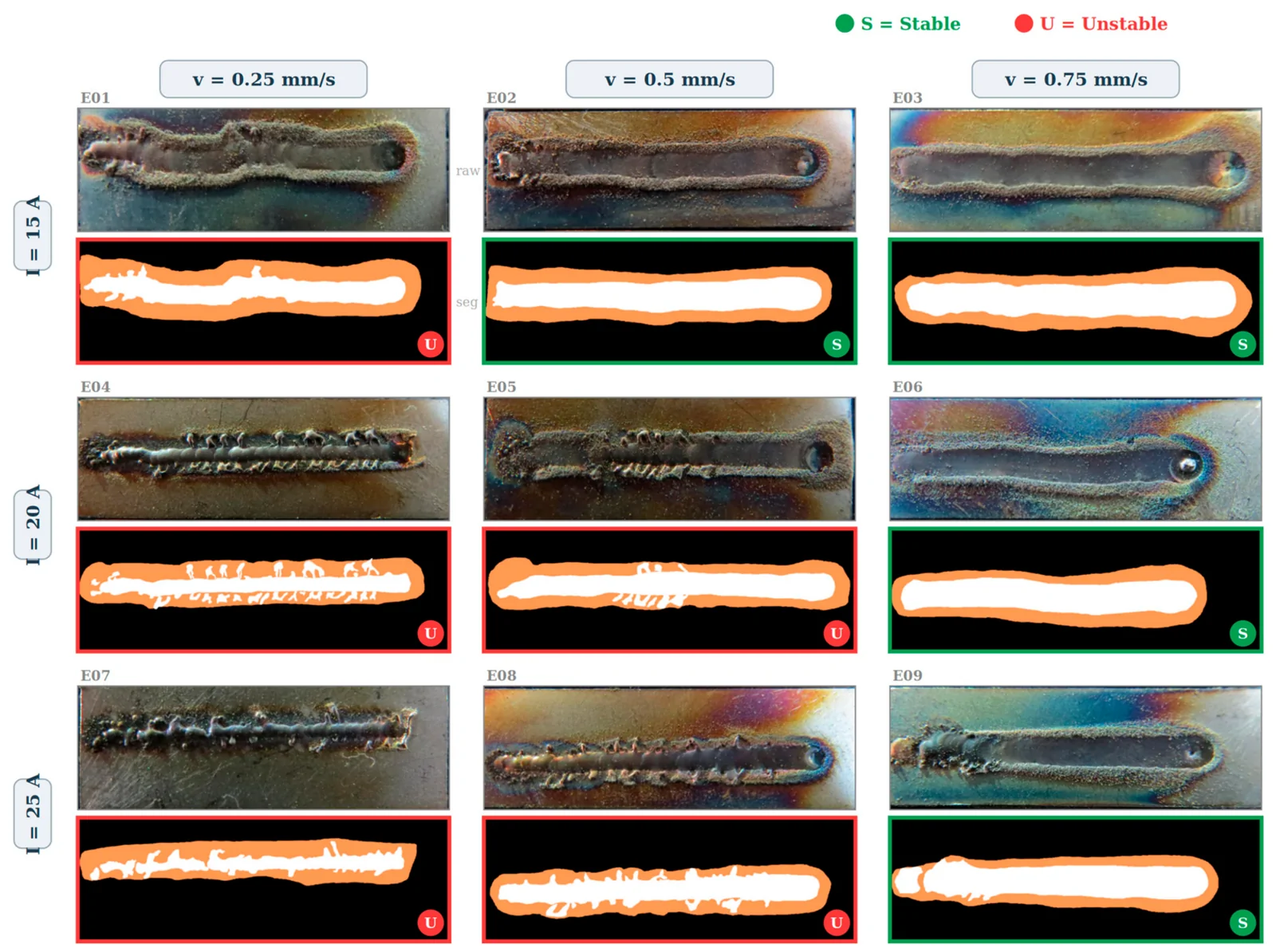
Combined effect of arc current and traverse speed on single-track morphology: 3 × 3 grid (fixed θ = 45°, d = 0.6 mm); within each cell, raw post-experiment image (**left**) and colour-coded segmentation (**right**). Frame colour indicates classification (green = stable, red = unstable). In the segmentation images the colour coding is as in Figure 3: melted region (white) and sintered region (orange).

The combined effect of current and traverse speed on track morphology is presented in Figure 8 for nine training experiments performed at fixed work angle (θ = 45°) and working distance (d = 0.6 mm). At fixed current, the increase in traverse speed narrows the track, makes the sintered band more distinct, and reduces discontinuities; at fixed traverse speed, the increase in current leads to local width fluctuations and agglomerations in the melt pool. This behaviour is consistent with the classical prediction that, at fixed speed, excessive heat input drives the melt pool into the Plateau–Rayleigh instability in the micro-TIG regime [12,25].

**Figure 9:**
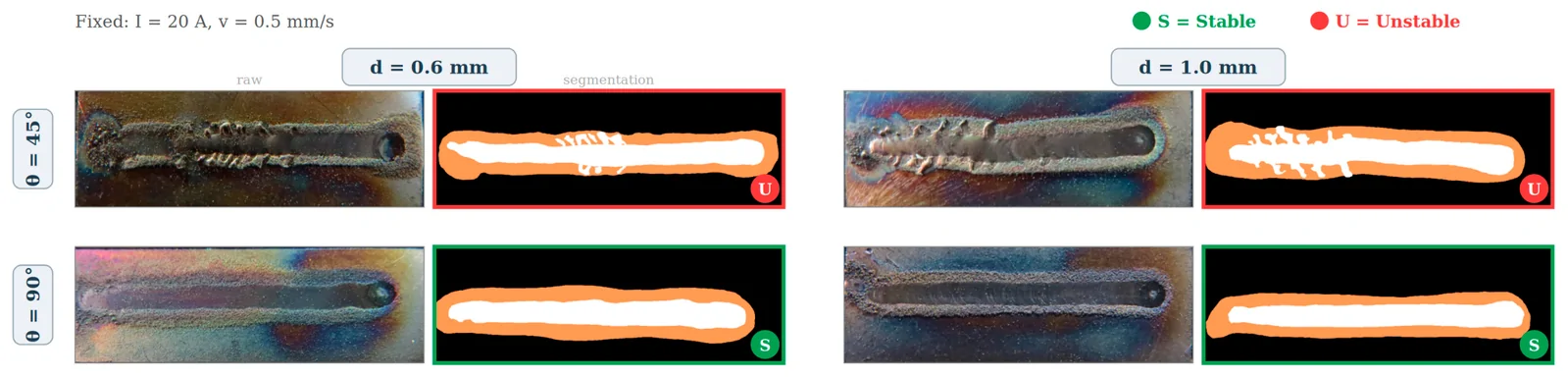
Effect of work angle and working distance on single-track morphology: 2 × 2 grid (fixed I = 20 A, v = 0.5 mm/s); within each cell, raw post-experiment image (**left**) and colour-coded segmentation (**right**). Colour coding of the segmentation images is as in Figure 3: melted region (white) and sintered region (orange).

The effects of the electrode work angle (θ) and working distance (d) on single-track morphology are shown in Figure 9 for four training experiments performed at fixed current (I = 20 A) and fixed speed (v = 0.5 mm/s). Increasing the work angle from 45° to 90° (that is, positioning the electrode perpendicular to the powder bed) produces a distinct stability gain in track morphology. This is interpreted as the perpendicular electrode configuration delivering arc-flux density to the bed more homogeneously and reducing lateral asymmetry. Increasing the working distance from 0.6 mm to 1.0 mm, on the other hand, lowers the energy-transfer efficiency, but does not severely compromise track stability even at θ = 90°.

### 3.2. Composite Quality Score and Classification Results

The weighted composite quality score S = 0.30·s_1_ + 0.30·s_2_ + 0.20·s_3_ + 0.20·s_4_ defined in Section 2.5 was applied to all 40 experiments. The classification results are summarised in Table 5.

Of the 36 experiments in the training set, 23 satisfied the condition S ≥ 0.75 and were classified as stable, while 13 fell below the threshold and were classified as unstable. All four experiments in the pure validation set were labelled unstable under the condition S < 0.75, with composite scores in the range 0.587 to 0.625. This score range represents the fragmented regime, which is unstable but not totally failed.

Sub-score analysis reveals which morphological metric dominates the instability. In the pure validation experiments, the continuity metric C = 1.0 is preserved; that is, the tracks appear uninterrupted along their length; however, the high agglomeration count (N_a_ = 12–53) and the relatively high linearity deviation (σ_cl_ = 0.17–0.24 mm) keep the score below the threshold. This quantitatively shows that, at speeds above the training upper bound (v > 0.75 mm/s), the melt pool transitions from the continuous melting regime to unstable droplet-ejection (spatter) behaviour [32].

**Figure 10:**
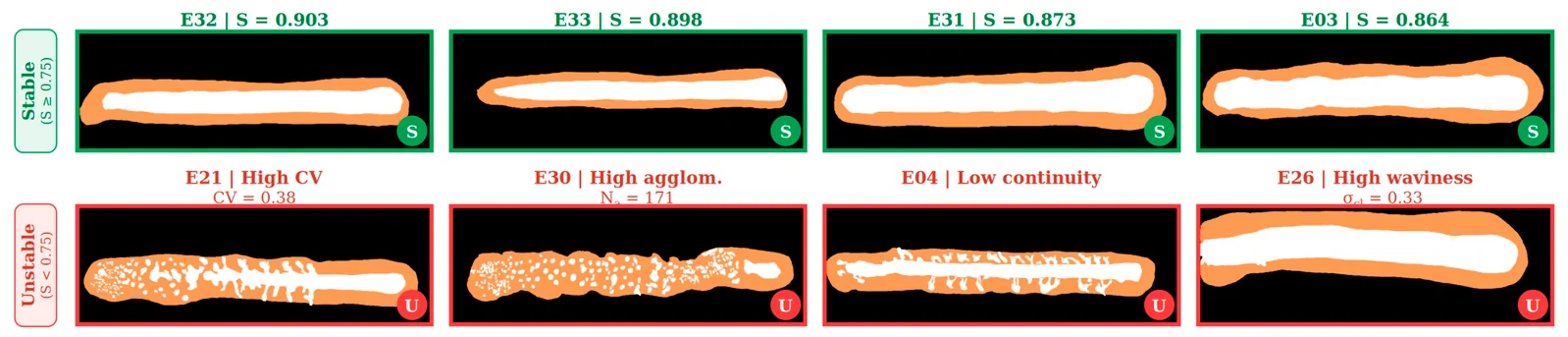
Quality filter outcomes: representative stable and unstable track regimes. Top row: four highest-scoring stable tracks (S ≥ 0.75). Bottom row: four unstable modes, namely high width variation (CV), high agglomeration count (N_a_), low continuity (C), and high linearity deviation (σ_cl_). Col-our coding of the segmented images is as in Figure 3.

Representative examples of stable and unstable track morphologies are presented in Figure 10. The upper row of the figure contains the four stable tracks with the highest composite score S (E32, E33, E31, E03; S ∈ [0.864, 0.903]); the lower row shows representative examples of four different instability modes (high CV, high N_a_, low C, high σ_cl_).

### 3.3. Morphological Analysis of the Pure Validation Set

**Figure 11:**
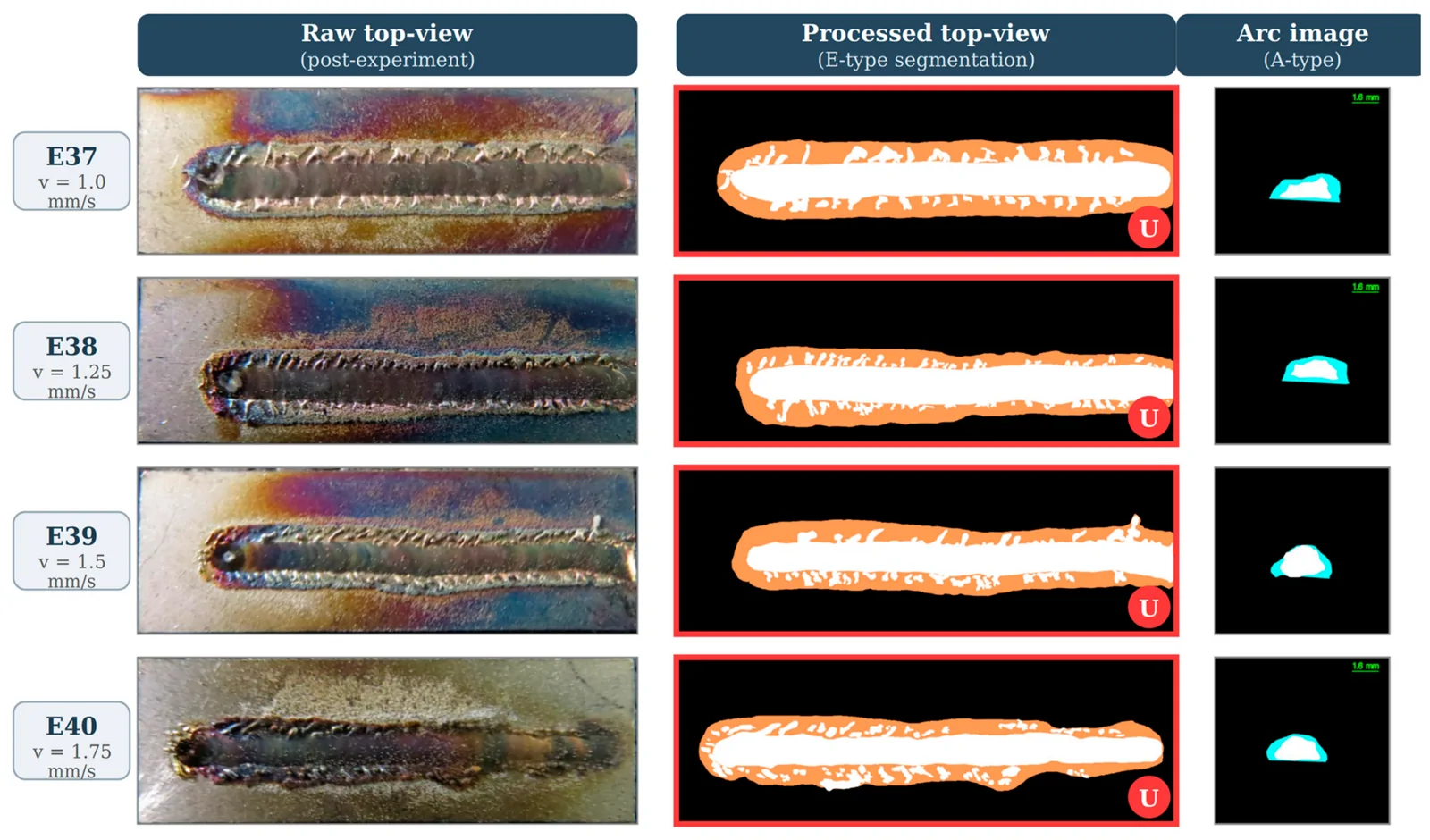
Pure validation set (E37–E40): speed extrapolation beyond the training range. Each row contains the raw post-experiment top-view image (**left**), the colour-coded E-type segmentation (**centre**), and the A-type arc image (**right**). Colour coding of the E-type segmentation is as in Figure 3.

The pure speed extrapolation set consists of four additional experiments scaled to 1.33–2.33× of the training upper bound (v = 0.75 mm/s): E37: v = 1.00 mm/s, E38: v = 1.25 mm/s, E39: v = 1.50 mm/s, E40: v = 1.75 mm/s, with fixed I = 25 A, θ = 45°, d = 0.6 mm. The top-view and arc images of these experiments along with their colour-coded segmentations are shown in Figure 11.

As the traverse speed increases from v = 0.75 mm/s to 1.75 mm/s, a narrowing of the track core width and a more distinct sintered band are observed in the top-view images. In the arc images, the spreading angle of the plasma column toward the bed (α_c_) appears to stabilise in the range of approximately 44–48°. The fact that all four experiments were performed under identical process conditions (fixed I, θ, d) enables the pure effect of traverse-speed scaling on morphology to be evaluated in isolation.

### 3.4. Pure Validation Performance of the PINN Model

The prediction accuracy of the PINN model on the pure speed extrapolation set was evaluated through four geometric features (D_q_, α_c_, w_HAZ_, d_core_). The w_iz_ output was not reported on the pure validation set because the quality filter classified all four validation points as unstable (Section 2.5.3 and Section 2.7.1). For the remaining four outputs, the MAPE and MAE metrics and their 95% confidence intervals obtained from a 1000-resample bootstrap analysis are presented in Table 6.

Model performance showed distinct differences among the four outputs. The mean MAPE was 4.25% for the arc core diameter D_q_, 8.06% for the heat-affected zone width w_HAZ_, and 6.29% for the arc cone angle α_c_. All three outputs are at the single-digit-percent MAPE level considered acceptable for engineering prediction in the single-track LPBF and PINN-AM literature [13,20].

For the track core width d_core_, the mean MAPE was 17.02%; this value is distinctly higher than the other three outputs. This elevation is related to the fact that the validation points are in extrapolation conditions scaled to twice the upper bound of the training range, together with the fact that d_core_ is geometrically not well defined on unstable tracks. The pure validation set being entirely classified as unstable indicates that the d_core_ measurement must also be interpreted in a quality-aware manner.

The upper bounds of the bootstrap confidence intervals remained below 10% for D_q_ and α_c_, and close to 10% for w_HAZ_. This shows that the model preserves significant predictive power under pure speed extrapolation; that is, under conditions that test the out-of-distribution generalisation capability that is one of the central claims of physics-informed learning.

**Figure 12:**
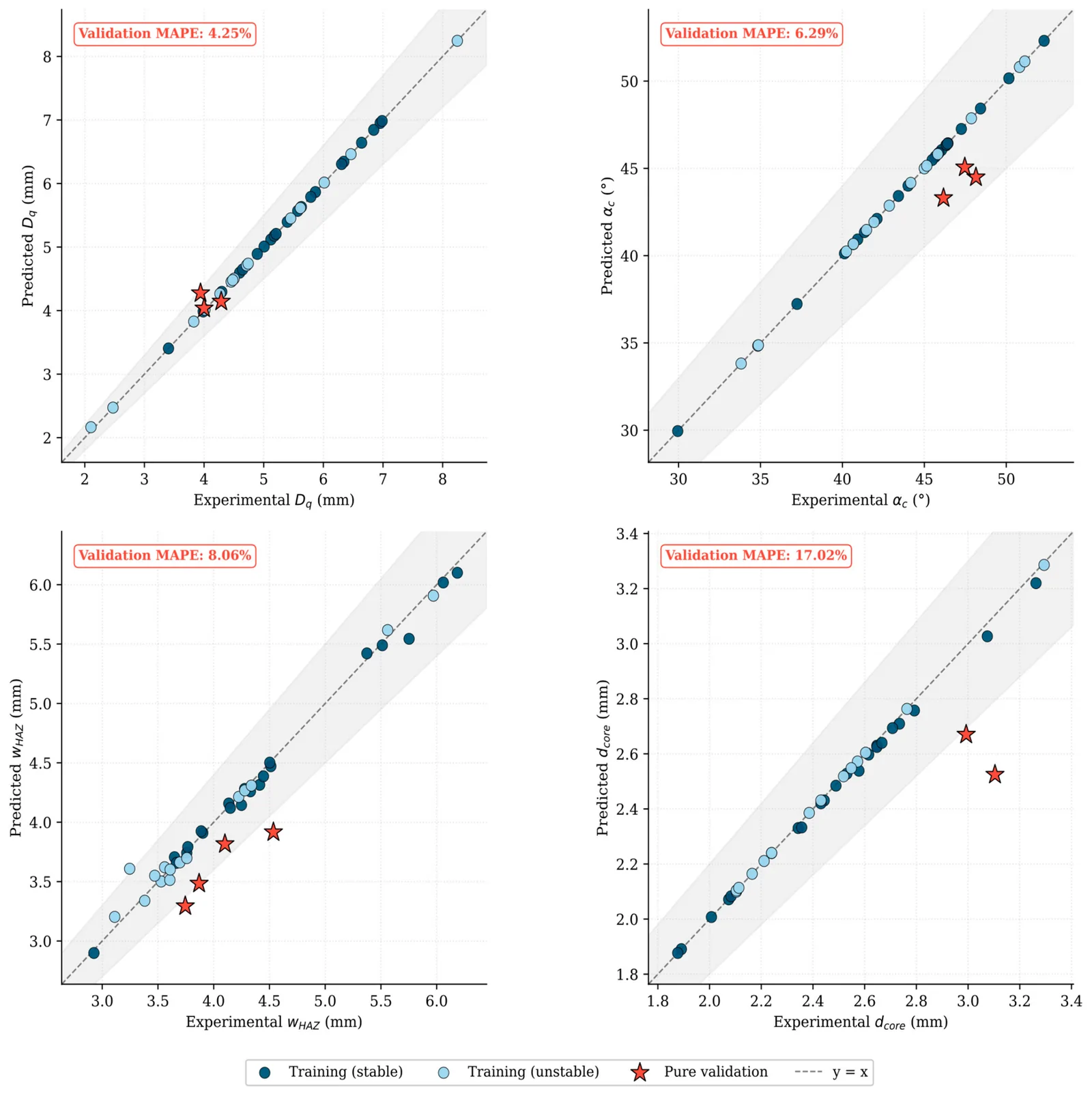
PINN parity plots: predicted versus experimental values for the four geometric features. Training points (blue circles, stable: dark/unstable: light) and pure validation points (red stars) are shown together with the y = x reference line and the ±10% band (grey shading).

The relation between the model predictions and the experimental measurements is presented as parity plots for the four outputs in Figure 12. The training points (blue circles) are gathered in a narrow band around the y = x line; the pure validation points (red stars) lie within or near the ±10% reference band. Only on the d_core_ panel do the validation stars fall outside the ±10% band, exhibiting behaviour consistent with the 17.02% MAPE value reported in Table 6.

### 3.5. Speed Extrapolation Curve

**Figure 13:**
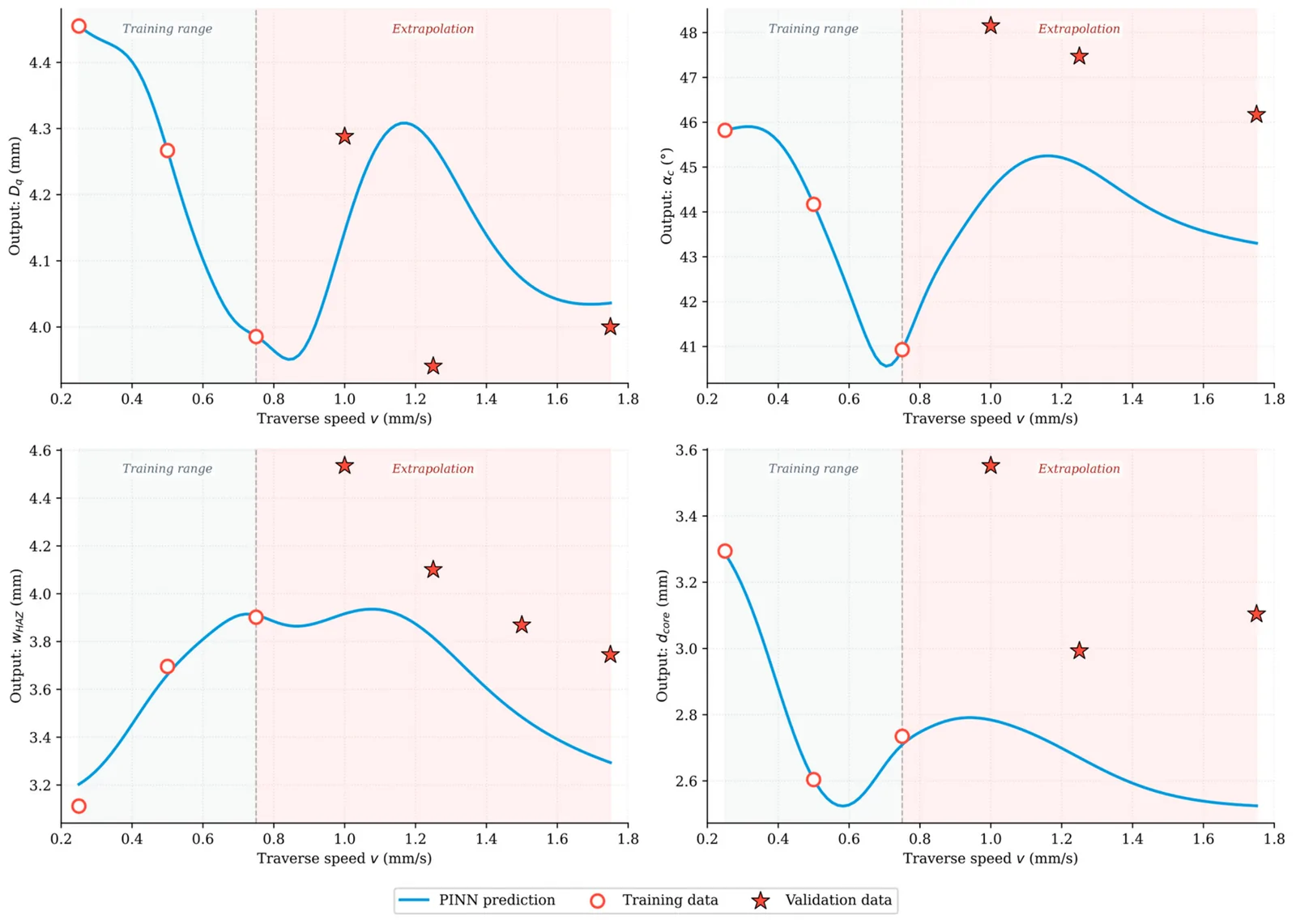
PINN prediction profile across pure speed extrapolation. Light background: training speed range (v = 0.25–0.75 mm/s); pink background: extrapolation range (v = 1.00–1.75 mm/s). Blue curve: PINN prediction profile at fixed (I = 25 A, θ = 45°, d = 0.6 mm); open circles: training points; red stars: pure validation points.

The PINN model’s prediction profile across the traverse speed was computed continuously at 100 points across v ∈ [0.25, 1.75] mm/s at fixed conditions (I = 25 A, θ = 45°, d = 0.6 mm) and overlaid on the training and validation points (Figure 13).

The prediction curves pass through the experimental points within the training range and exhibit physically plausible peak–valley behaviour in the extrapolation region. The D_q_ and α_c_ outputs remain close to experimental values at the extrapolation points; the w_HAZ_ output shows a controlled decreasing trend; for the d_core_ output, the validation stars lie above the prediction curve, visually demonstrating that the model exhibits comparatively lower performance for this output under speed extrapolation.

### 3.6. Learned Ayrton Voltage Sub-Module

**Figure 14:**
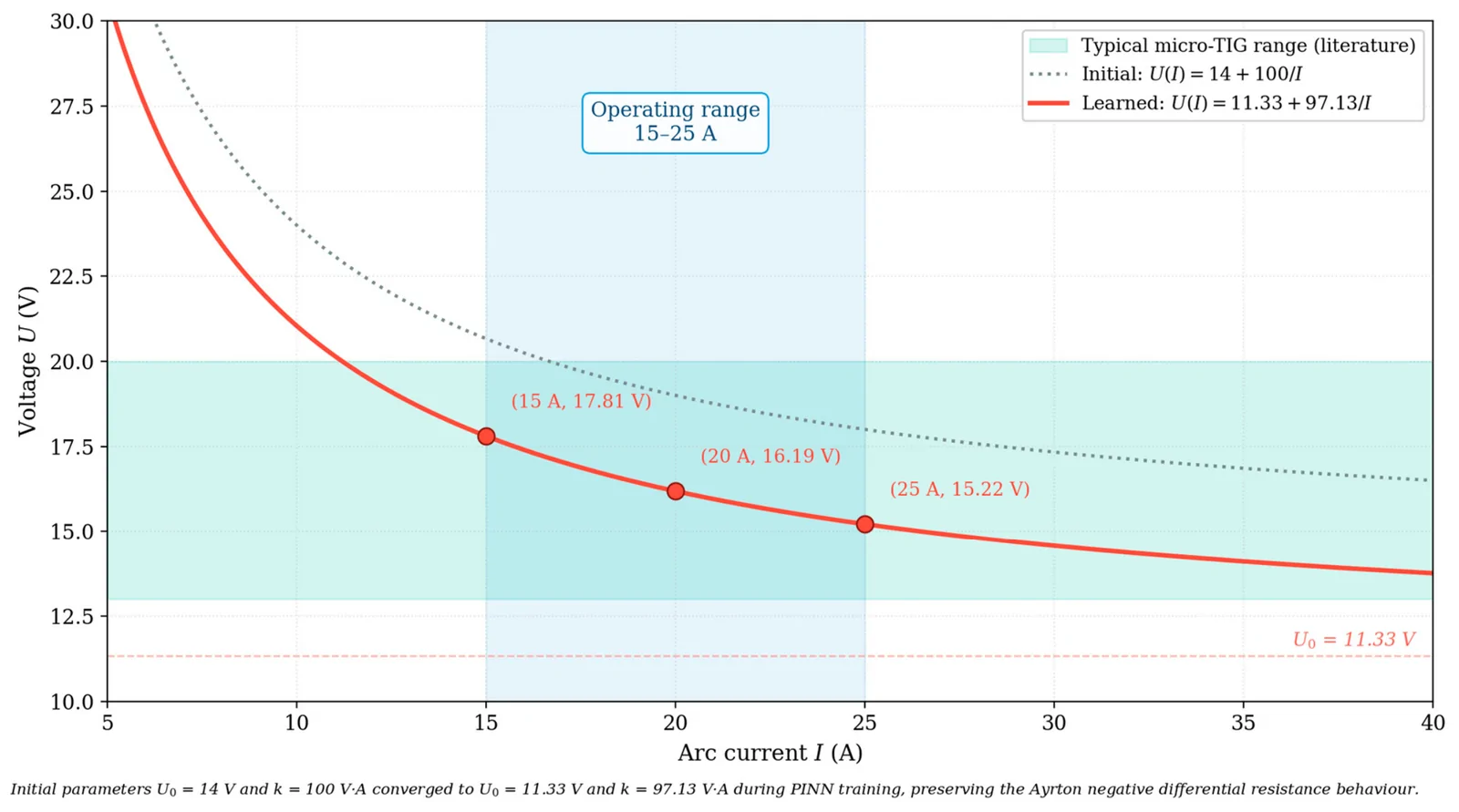
Ayrton voltage sub-module U(I) = U_0_ + k/I learned by the PINN. Red curve: learned U(I) = 11.33 + 97.13/I; grey dashed curve: U(I) = 14 + 100/I obtained from initial values; green background: indicative voltage range for short low-current argon TIG arcs (see Table 7); blue background: operating current range (15–25 A).

During training of the PINN model, the two learnable parameters of the Ayrton voltage sub-module defined in Section 2.7.5 (U_0_ and k) were updated simultaneously with the network weights. Starting from initial values U_0_ = 14.00 V and k = 100.00 V·A, the parameters converged to U_0_ = 11.33 V and k = 97.13 V·A at the end of training. The trained voltage function is tabulated for the current range in Table 7 and presented graphically in Figure 14.

The learned voltage curve is qualitatively consistent with the physical behaviour of low-current TIG arcs reported [21]. More importantly, the monotonically decreasing behaviour of voltage with current (U(15 A) = 17.81 V > U(25 A) = 15.22 V) captures correctly the negative differential resistance behaviour, which is the fundamental physical expression of the Ayrton equation.

The voltage sub-module is learned indirectly through the physics loss term L_phys_ (Section 2.7.2), not from direct experimental voltage measurements. The distinct deviation of the learned parameters from their initial estimates (U_0_: 14.00 → 11.33 V; k: 100.00 → 97.13 V·A) indicates that the physics term supplies a genuine gradient to these two parameters rather than leaving them at their initial values. A control experiment supports this reading: when the physics loss is switched off and the two parameters are excluded from weight decay, they remain exactly at their initial values of 14.00 V and 100.00 V·A, whereas with the physics loss active they move to the values reported above. It should be noted, however, that the magnitude of this displacement is not by itself a measure of identification, since the scaling factor of the heat-conduction residual is refitted at every optimisation step and the residual is therefore invariant to a multiplicative rescaling of the predicted width. The learned parameters should accordingly be read as physically plausible rather than as uniquely determined; this point is developed further in Section 4.3.

The voltage box-constraint loss L_volt_ (8 ≤ U ≤ 30 V) successfully enforced the learned values to remain within a physically reasonable range; the condition k > 0 preserved the monotonically decreasing behaviour of voltage with current. Within the operating current range (15–25 A), the learned U(I) curve is located in the central region of this indicative range.

### 3.7. Geometric Consistency Among Outputs

The PINN model’s outputs on the pure validation set were examined against the three geometric constraints defined in Section 2.7.3. In all four validation experiments, the conditions w_HAZ_ > w_iz_ (HAZ-track ordering, g_1_), D_q_ > d_core_ (arc core-track core ordering, g_2_), and d_core_ ≤ w_iz_ ≤ 2.5·d_core_ (ratio constraint, g_3_) were examined for the predicted values. The two ordering constraints g_1_ and g_2_ were satisfied at all four validation points. The lower bound of the ratio constraint g_3_ was not satisfied: the predicted d_core_ exceeded the predicted w_iz_ at every validation point. This outcome is consistent with the measured data rather than at variance with it because d_core_ is defined in Section 2.4.2 as the mean of the column-wise extent between the uppermost and the lowermost track pixels and therefore spans intra-track gaps, whereas w_iz_ is an areal quantity that excludes them. In the measured dataset, d_core_ exceeds w_iz_ in 33 of 39 experiments, so the lower bound as originally coded imposed the opposite of the ordering that the two definitions produce. The bound is retained here in its original form for consistency with the trained model, and its correction is identified in Section 5 as an item for future work. The behaviour of the two ordering constraints nevertheless shows that the soft-constraint formulation of the geometric loss term L_geom_ is able to preserve physical consistency among the outputs even outside the training range.

Checking the partial derivatives of the model with respect to the current shows that both ∂w_iz_/∂_I_ and ∂d_core_/∂_I_ remain positive across the training range and at the validation points. This is quantitative evidence that the monotonicity loss L_mono_ guides the network’s local rise–fall behaviour among the training examples in a physically reasonable direction.

### 3.8. Comparison with Purely Data-Driven Models and Ablation of the Framework Components

To determine whether the predictive performance reported above originates from the physics constraints, from the shared multi-task architecture, or from the neural network alone, four configurations were trained on the identical dataset, with the identical optimiser settings and for the identical number of epochs. Configuration A uses five independent single-output networks with the data-fit loss only. Configuration B adds the physics terms to those independent networks. The geometric consistency term cannot be defined in this setting, since it couples outputs that independent networks do not share, and it is therefore omitted. Configuration C is the shared-trunk multi-task network with the data-fit loss only. Configuration D is the complete model. Each configuration was trained with three random seeds and the mean validation MAPE across the four measurable outputs is reported. Five classical regression models were trained on the same inputs and targets for reference. The results of all configurations are collected in Table 8. For reference, the single-seed complete model used in the remainder of the paper (seed 42; Table 6 and Table 9) attains 8.90 per cent on the same metric; the difference from the three-seed average of 10.58 per cent reflects the seed spread discussed in Section 5.

Two results follow. First, in aggregate the shared multi-task trunk is the larger contributor. Moving from configuration A to configuration C reduces the mean error by 11.4 percentage points, whereas adding the physics terms on top of that architecture accounts for a further 1.9 points. This aggregate figure requires qualification, because it is driven almost entirely by one output. Per output, the validation errors of configurations A, C and D are 7.56, 10.52 and 10.09 per cent for the heat-affected zone width; 57.10, 13.22 and 6.24 per cent for the arc core diameter; 21.03, 13.83 and 16.23 per cent for the track core width; and 9.80, 12.48 and 9.76 per cent for the cone angle. The arc core diameter therefore accounts for essentially all of the aggregate gain, independent networks are the best configuration for the heat-affected zone width, and the physics terms degrade the track core width relative to the shared trunk alone. The benefit of the shared architecture is thus specific to the outputs that are poorly determined in isolation rather than uniform across the output set. The physics terms applied without the shared trunk yield only 0.6 points, which is expected, since the geometric consistency term is the largest of the physics weights and cannot act at all when the outputs are predicted by separate networks. The contribution of the physics formulation is therefore real but conditional on the multi-output architecture, and the framework should be understood as one in which the architecture and the physical constraints act together rather than as one in which the constraints alone drive the performance.

Second, the classical regression models are competitive with, and in the aggregate somewhat better than, the proposed model on this validation set. This comparison requires careful reading. The four validation experiments vary in a single direction, traverse speed, at fixed current, work angle and working distance, and the corresponding target values are narrowly distributed. The arc core diameter, for example, takes the three values 4.288, 3.941 and 4.000 mm, with a standard deviation of 0.152 mm. On a target of such small dispersion, a model that simply reproduces a value near the training boundary attains a low percentage error without extrapolating at all, which is precisely what a random forest does, since it cannot by construction predict outside the range of its training targets. The comparison therefore does not establish that the classical models extrapolate better. What it establishes is that a four-point validation set of this shape lacks the statistical power to separate the models. What distinguishes the proposed model in this comparison is not aggregate accuracy but that it delivers five outputs from a single network while maintaining the physical ordering relations among them, and that it recovers a voltage relation that was never measured. The classical models offer neither property, and each of them requires a separate fit per output with no guarantee of mutual consistency.

### 3.9. Sensitivity of the Model to the Physics Loss Weights

The relative weighting of the loss components was examined by scaling each weight in turn while holding the remaining three at their nominal values. Relative to the nominal mean validation MAPE of 8.90 per cent, removing the heat-conduction term raises the error to 11.88 per cent, halving its weight gives 15.13 per cent and doubling it gives 11.41 per cent; removing the geometric consistency term gives 9.92 per cent, halving it 9.48 per cent and doubling it 10.57 per cent; removing the monotonicity term gives 14.77 per cent, halving it 9.28 per cent and doubling it 9.44 per cent. Scaling the box constraint on the arc voltage has no effect whatsoever. Zero, half and twice the nominal value all return 8.90 per cent, because the learned voltage remains inside the admissible interval throughout training and the constraint is therefore never active. It is retained as a safeguard rather than as a contributing term. Three observations follow. Every perturbation of an active weight degrades performance, so the nominal values sit at a local optimum with respect to the three terms that are active. The monotonicity and heat-conduction terms carry the most information, their removal costing 5.87 and 2.98 percentage points, respectively, while the geometric term contributes 1.02 points. At the same time, the response to the heat-conduction weight is not monotonic, with the halved weight performing worse than the removed weight, which indicates that at this dataset size the optimisation landscape is sensitive to the interaction between the weighted terms. The same non-monotonic response is seen for the monotonicity weight, where the halved weight also performs better than the doubled one. The nominal values should therefore be regarded as a working choice within a broad and shallow optimum rather than as a finely tuned one. This sensitivity is one reason why the limitations in Section 5 emphasise the size of the dataset.

### 3.10. Extended Error Metrics, Convergence Behaviour and Cross-Validation

The reported errors are extended here to a fuller set of metrics, and the two questions of convergence and of in-distribution generalisation are addressed separately from the extrapolation result of Section 3.4. The extended metrics are given in Table 9.

**Figure 15:**
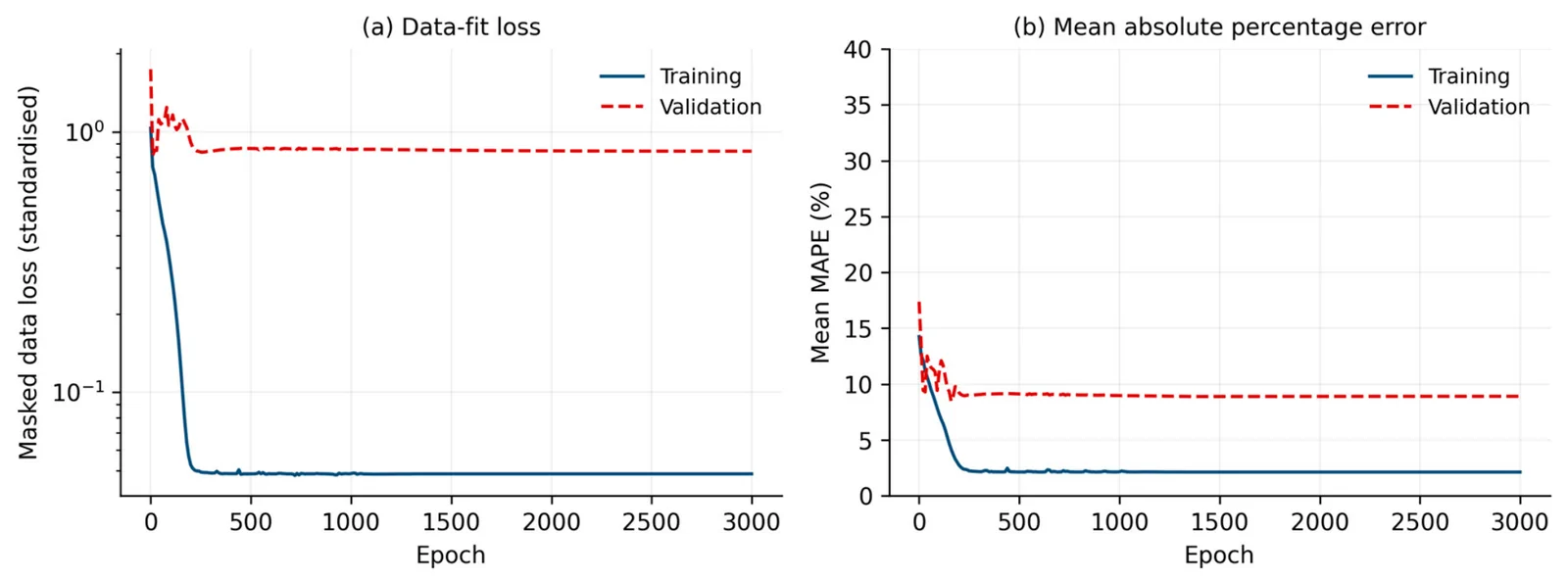
Convergence of the complete model. (**a**) Masked data-fit loss on the training and validation sets, logarithmic ordinate. (**b**) Mean absolute percentage error over the measurable outputs.

The negative coefficients of determination on the validation set require explanation, since they might otherwise be read as indicating that the model performs worse than the mean of the targets. The four validation experiments vary in a single direction and the targets are consequently of very small dispersion. The arc core diameter takes the values 4.288, 3.941 and 4.000 mm, a standard deviation of 0.152 mm, and the cone angle varies over less than two degrees. The denominator of the coefficient of determination is that dispersion, so a modest absolute error yields a large negative value irrespective of the model. The coefficient is therefore reported for completeness but is not informative for this validation design, and the absolute and percentage errors should be used instead. The training coefficients, by contrast, are close to unity for four of the five outputs, which reflects the parameter-to-sample ratio discussed in Section 5 rather than predictive skill. The convergence of the training and validation losses is shown in Figure 15.

The convergence behaviour bears directly on the question of overfitting. The training loss falls steeply during the first 250 epochs and is essentially flat thereafter. The validation loss stabilises over the same interval and then remains flat for the remaining 2750 epochs without any upward drift. There is thus no progressive overfitting in the usual sense, in which validation error deteriorates while training error continues to fall. What the curves do show is a persistent offset. The training data-fit loss settles at 0.049 against 0.844 on the validation set, and the corresponding mean errors are 2.12 and 8.90 per cent. This offset is established early and is attributable to the distribution shift between the two sets rather than to prolonged training, and the training schedule could be shortened considerably without loss of accuracy.

Leave-one-out cross-validation was performed to assess in-distribution generalisation, which the extrapolation set cannot measure. Each of the 36 training conditions was held out in turn, the model retrained on the remaining 35, and the held-out condition predicted. The resulting errors are 14.07 per cent for the heat-affected zone width, 22.37 per cent for the arc core diameter, 14.83 per cent for the track core width and 16.17 per cent for the cone angle, giving a mean of 16.86 per cent across the four outputs; the areal track width, evaluated on the 21 stable tracks that enter the areal-width target (Section 2.5.3), gives 19.86 per cent. These figures are approximately twice the error obtained on the extrapolation set, and the discrepancy is instructive rather than contradictory. The extrapolation set consists of four points whose targets are narrowly distributed, whereas cross-validation removes conditions from across the whole design, including those at the corners of the process window where no neighbouring condition supports the prediction. The worst fold, averaged over its outputs, reaches 57.2 per cent, which confirms that a small number of poorly supported conditions dominates the average. Cross-validation therefore provides the more conservative and, for practical purposes, the more relevant estimate of the error to be expected at an untried parameter combination inside the process window.

### 3.11. Sensitivity of the Predictions to the Process Parameters

The partial derivatives of each output with respect to each input are available directly from the trained network by automatic differentiation, which permits the influence of the four process parameters to be compared without any surrogate attribution method. Table 10 reports the mean absolute derivative over the training conditions, computed on the normalised inputs and expressed as a percentage of the total for each output.

Traverse speed is the dominant parameter for every output, accounting for between 52 and 61 per cent of the total sensitivity, followed by arc current at approximately 20 per cent. This ordering is what the energy balance requires. The energy delivered per unit length is proportional to the current and inversely proportional to the speed, so over a sevenfold speed range and a range of current of less than a factor of two, speed is necessarily the stronger lever. The result is therefore not an independent physical discovery but a consistency check, and it indicates that the network has learned a relationship of the expected form rather than an arbitrary interpolation of the training points. Work angle and working distance contribute least, between 8 and 16 per cent, consistent withhaving been sampled at two levels only.

### 3.12. Comparison with an Analytical Heat-Source Model and Computational Cost

To place the framework against an established analytical approach, the classical Rosenthal solution for a moving point source on a semi-infinite body was applied to the same conditions. The maximum width of the liquidus isotherm was computed numerically for each training condition, with the effective energy input defined exactly as in the heat-conduction residual of Section 2.7.2. The effective thermal conductivity of the powder bed is not known independently, and the comparison was therefore made in two ways. With the conductivity fixed at 13.0 W m^−1^ K^−1^, close to the bulk value for 316L, the analytical solution yields a mean absolute percentage error of 44.7 per cent on the 21 stable training tracks that enter the areal-width target, with a root mean squared error of 1.11 mm and a correlation of 0.35 between predicted and measured widths. Treating the conductivity instead as a single free parameter fitted to the same training data gives the analytical model the most favourable comparison available. The optimum lies at 20.0 W m^−1^ K^−1^ and the error falls to 19.6 per cent, with a root mean squared error of 0.54 mm and a correlation of 0.33. The corresponding training error of the complete model on the same quantity is 10.1 per cent, so even under the most favourable treatment the analytical solution remains about twice as inaccurate.

The weak correlation is more informative than the error magnitude, and it is essentially unchanged between the two treatments, which shows that the discrepancy is not a matter of scale that a different conductivity would absorb: the analytical model does not reproduce the observed trends across the parameter space. The reason is visible in the predictions themselves. Over the traverse speeds sampled here, 0.25 to 0.75 mm s^−1^, the Rosenthal width is almost independent of speed, varying by less than one per cent between the lowest and highest speed at fixed current, whereas the measured widths vary appreciably. The sampled conditions lie deep in the low-Péclet, quasi-stationary regime of the analytical solution, in which the width is governed by the current alone. The speed dependence that the measurements show, and that the network learns, therefore lies outside the reach of the point-source description at these speeds. Three of its assumptions are violated here. The medium is not semi-infinite, since the powder layer is 150 μm thick on a substrate of different conductivity. The thermal properties are not constant, since the effective conductivity of the loose powder differs from that of the consolidated track by roughly an order of magnitude and changes as the material melts. The source is not a point, since the arc column has a finite diameter comparable to the track width, as the measured arc core diameters in Table 6 show. A finite-element treatment could relax the first and the third of these but would require the temperature-dependent effective conductivity of the powder bed, which was not measured in this study. This comparison therefore does not establish that analytical or numerical thermal models are inadequate in general. In the low-current powder-bed regime studied here they require material data that a purely geometric experimental campaign does not provide, and that is the specific gap the present approach addresses.

Regarding computational cost, training the complete model for 3000 epochs on the full batch of 36 conditions was carried out on an Apple M5 processor with 10 logical cores and 24 GB of unified memory, under macOS 26.5.1 with Python 3.14.2 and PyTorch 2.13.0, and required 3.1 s. No graphics processing unit was used at any stage, and none is necessary at this problem size. Inference is a single forward pass through 13,767 parameters and required 0.0165 ms per prediction, averaged over 10,000 repetitions after a warm-up phase. This returns all five geometric features simultaneously. The contrast with conventional simulation is accordingly large. A transient finite-element thermal analysis of a single track typically requires minutes to hours of computation for one parameter combination, so that mapping a four-dimensional process window at any useful resolution is impractical, whereas the present model evaluates thousands of parameter combinations in well under a second; at the measured inference time, five thousand evaluations of the process window require less than 0.1 s. It follows that computation is not the obstacle to in-process use of this model. As Section 3.13 argues, the limiting factor is predictive accuracy on the less well determined outputs rather than inference speed. The cost of the approach lies elsewhere, in that the model must first be trained on experimental data and does not transfer to a new material or electrode configuration without new experiments.

### 3.13. Suitability of the Achieved Accuracy for Process Control

Whether the accuracy reported here is sufficient for in-process control depends on the tolerance that the application places on track geometry. For powder-bed processes the practical requirement is usually expressed through the overlap between neighbouring tracks. Hatch spacing is set as a fixed fraction of the track width, and lack-of-fusion porosity appears when the actual overlap falls below the design value. With a hatch spacing conventionally set between 60 and 80 per cent of the nominal width, a prediction error of 10 per cent in width consumes a substantial part of the margin that separates a sound overlap from an unfused one, while an error of 20 per cent would consume it entirely. On that criterion the arc core diameter, predicted to 4.3 per cent on the extrapolation set, is within a useful range; the track core width, at 17.0 per cent on the extrapolation set and 14.8 per cent under cross-validation, is not. The honest conclusion is that the present accuracy supports offline parameter selection and process window mapping, where the model narrows the region that must be verified experimentally, but does not yet support closed-loop control, which would in addition require the per-prediction uncertainty discussed in Section 5 rather than a point estimate. It should also be noted that the errors quoted here are obtained from a single deposition pass under laboratory conditions and would need to be re-established on a production system before any control application.

## 4. Discussion

In this section, the experimental and numerical findings reported in Section 3 are discussed in the context of the relevant literature. The discussion proceeds along six axes: (i) the positioning of the PBAAM process window relative to the authors’ previous studies, (ii) the comparison of the PINN model’s prediction accuracy with the physics-informed learning literature, (iii) the interpretation of the learned Ayrton voltage sub-module in the context of arc physics, (iv) the signature of the Plateau–Rayleigh instability in the composite quality score, (v) the physical meaning of geometric consistency among outputs, and (vi) the distinctive contribution of pure speed extrapolation to the physics-informed learning literature.

### 4.1. Comparison with Previous Studies: The PBAAM Process Window

When the findings of this study are compared directly with the authors’ two previous PBAAM publications, it can be seen that the process-window map has been quantitatively extended. Balcı and Alibeyoğlu [6] performed inclined-bed (0–0.4 mm thickness gradient) experiments with a 1.6 mm WL20 electrode on 316L stainless steel and reported that the melt-pool width grew at every measurement point as the current was increased; however, narrowing in the trailing region of the track was observed for high-current/high-speed combinations. In the present study, the same qualitative behaviour (current → wider track, speed → narrower track) was observed in 36 experiments performed at a fixed layer thickness of 150 μm (Figure 8); this agreement shows that the fundamental thermal behaviour of the PBAAM process family is preserved independently of layer thickness.

Çelikel and Balcı [7], using a fixed 0.6 mm tungsten electrode diameter, mapped the Continuous-Stable, Transitional and Fragmented morphological regimes via image-based topological analysis across 17 different process conditions. Their study covered a three-stage systematic parameter sweep over 10–30 A welding current, 0.3–0.8 mm electrode-to-powder-bed stand-off distance and 2–8 mm/s scanning speed, and proposed a deterministic classification framework based on connected-component count (N) and the width coefficient of variation (CV). The present study examines 36 training experiments at lower current/speed (15–25 A, 0.25–0.75 mm/s) in the micro-TIG regime with four independent parameters (additionally including electrode work angle and a smaller working distance) of the same author; 23 of these experiments are classified as stable and 13 as unstable. Together, the two studies therefore constitute complementary data layers for mapping the PBAAM process window across both the macro-current/speed regime [7] and the micro-current/speed regime (present study).

Taken together, these three studies constitute a systematic infrastructure for the macro-experimental map of the PBAAM process window: Balcı and Alibeyoğlu [6] address the effect of layer thickness; Çelikel and Balcı [7] address the effects of electrode stand-off distance and macro-current/speed; and the present study addresses the multi-output prediction model under micro-current/speed.

### 4.2. Comparison of Prediction Accuracy with the PINN-AM Literature

The MAPE values obtained on the pure speed extrapolation set (D_q_: 4.25%; α_c_: 6.29%; w_HAZ_: 8.06%; d_core_: 17.02%) are in a range comparable with the benchmarks reported in the single-track LPBF and PINN-AM literature. Hosseini et al. [20] reported a mean absolute error below 5% for the temperature field and melt-pool dimensions in single-track LPBF via parametric physics-informed neural networks (relative to the finite element reference solution), while Sajadi et al. [13] proposed the first physics-informed online-learning framework for temperature-field prediction in metal AM, showing that PINNs adapt particularly well to changes in process parameters, geometry, and material in predicting temperatures of the heat-affected zone and the melt pool. McGowan et al. [42] proposed a physics-informed convolutional neural network (PI-CNN) with customised loss functions for porosity detection in laser metal deposition (LMD), reporting approximately 94% classification accuracy on the test set (this work targets a classification objective rather than a continuous regression with MAPE metric). The three main outputs of the present study (D_q_, α_c_, w_HAZ_) remain at single-digit MAPE and show that PBAAM can attain a prediction accuracy comparable with that of LPBF/DED-based PINN applications.

The 17.02% MAPE for the track core width (d_core_) is distinctly higher than the other three outputs. This elevation can be explained by two factors. First, all four points of the pure validation set were classified as unstable by the quality filter; second, the d_core_ feature could not be geometrically defined consistently on unstable tracks due to local narrowing and agglomeration. Liu et al. [43], examining process–structure–property relationships in metallic AM with an interpretable machine-learning approach, showed that prediction errors can rise markedly in the unstable track regime; the d_core_ error in the present study is qualitatively consistent with this literature finding.

The upper bounds of the bootstrap confidence intervals remain below 10% for D_q_ and α_c_ and close to 10% for w_HAZ_, showing that the model preserves significant predictive power under pure speed extrapolation even with limited sample size. This empirically supports the thesis of Karniadakis et al. [15], one of the central claims of physics-informed learning, that out-of-distribution generalisation capability can be achieved even under small-sample conditions. Within the taxonomy of Faegh et al. [21], which distinguishes loss-function modification, architecture modification and multi-modal learning, the present study combines all three categories: the loss function carries the Rykalin-type heat-conduction, geometric-consistency and monotonicity constraints; the Ayrton voltage relation is embedded as an architectural sub-model; and the targets measured from the two imaging modalities are joined under a single shared trunk.

A qualification is necessary before these figures are compared. The prediction tasks are not equivalent. The studies cited above predict temperature fields or other simulation-derived quantities and are typically validated against finite element reference solutions, on datasets that can be generated in whatever quantity the study requires. The present work predicts experimentally measured geometric features from a physical dataset of 36 training conditions and 4 extrapolation conditions. An error measured against a simulation reference and an error measured against a physical measurement do not carry the same noise floor, since the latter includes the variability of the process itself and of the image-based measurement chain. Dataset sizes and validation strategies differ correspondingly. The comparison should therefore be read as the qualitative observation that physics-informed formulations retain useful accuracy under distribution shift in both settings, and not as a ranking of the reported error magnitudes.

### 4.3. Physical Interpretation of the Learned Ayrton Voltage Sub-Module

The Ayrton voltage function U(I) = 11.33 + 97.13/I to which the PINN model converged during training without any direct voltage measurement is important in three principal aspects of micro-TIG arc physics. First, the monotonically decreasing behaviour of the voltage with current (U(15 A) = 17.81 V, U(20 A) = 16.19 V, U(25 A) = 15.22 V) is qualitatively consistent with the negative differential resistance behaviour defined by Ayrton’s classical carbon-arc experiments in 1902 [23]. Second, the fact that the learned values are on the order of tens of volts and decrease monotonically with current, consistent with the arc-column behaviour reported in numerical analyses of low-current TIG welding [22] confirms that the model has settled into a physically plausible parameter space. Third, the marked deviation of the learned parameters from their initial values (U_0_: 14.00 → 11.33 V; k: 100.00 → 97.13 V·A) shows that the physics term supplies a genuine gradient to these parameters, since they remain at their initial values when that term is removed. This does not, however, establish that the voltage function is uniquely identified from the geometric data. The heat-conduction residual is evaluated against a proportionality factor that is refitted at each optimisation step, so the residual is invariant under a multiplicative rescaling of the predicted track width; consequently only the shape of U(I), and not its absolute level, is constrained by the data. Over the sampled current interval of 15 to 25 A the form U_0_ + k/I is moreover close to linear, which further limits the separation of the two parameters. The learned relation should therefore be interpreted as one member of a family of voltage functions compatible with the observed geometry, selected within that family by the box constraint and by the initial values, rather than as a unique inverse solution.

This result is a concrete instance of one of the central theoretical claims of Raissi et al. [14], namely the indirect parameter estimation capability of physics-informed neural networks. In classical inverse problem approaches, the estimation of a physical parameter requires direct or at least indirect measurement of that parameter; in the PINN framework, by contrast, the parameter can be inferred indirectly via the physical constraint on geometric feature measurements. Cai et al. [24] presented a similar application of this approach in fluid mechanics, showing that the Reynolds number can be inferred only from velocity field data without direct measurement. The Ayrton voltage sub-module in the present study is an application of this methodology in the metallic AM context.

The physical plausibility of the learned U(I) curve is also consistent with the principal predictions of micro-TIG arc plasma physics. Pang et al. [22] showed for low-current TIG welding via a two-temperature numerical model that the arc voltage rises steeply as the current decreases, driven principally by the increase in the near-cathode voltage drop, while the arc column contracts at low current. The k = 97.13 V·A coefficient in the present study is the empirical measure of this inverse dependence of voltage on current. As noted above, the small displacement of this coefficient from its initial value is not by itself evidence of identification, since only the shape of U(I) and not its absolute level is constrained by the geometric data. The convergence of the asymptotic value of *U*_0_ to 11.33 V can be interpreted as a minimum sustaining voltage corresponding to the sum of the voltage drops at the anode and cathode regions of the plasma column at non-zero current limits [41].

### 4.4. Plateau-Rayleigh Instability and the Quality Filter

The composite quality score’s classification of the four experiments in the pure validation set as unstable in the range 0.587–0.625 indicates that, at speeds beyond the upper bound of the training range (v = 0.75 mm/s), the melt pool enters a characteristic instability mode. While the continuity metric C = 1.0 is preserved (i.e., the tracks appear continuous along their length), the agglomeration count rises to N_a_ = 12–53 and the linearity deviation to σ_cl_ = 0.17–0.24 mm. This behavioural signature is in quantitative agreement with the Plateau–Rayleigh instability described in the classical LPBF single-track literature.

The Plateau–Rayleigh instability is the tendency of the melt pool to break into independent droplets under the influence of surface tension; it becomes dominant when the ratio of melt-pool length to width exceeds a critical threshold value and leads to droplet detachment and periodic width fluctuations. Classical LPBF single-track studies showed that at sufficiently high scanning velocities the elongated melt pool becomes subject to balling, and that this behaviour is explained by the Plateau–Rayleigh capillary instability of the melt pool [12,25]. Yadroitsev et al. [12] established from single-track experiments that the stability of the deposited track is governed by the geometry of the melt pool, the transition to the capillary-instability regime being expressed as a threshold on the ratio of melt-pool length to width. Boutaous et al. [31] reached a consistent conclusion from the modelling side, showing with a coupled thermal and discrete-element model of the laser melting process that the formation of balling droplets is governed by the process parameters and material properties that set the melt-pool behaviour.

The behaviour observed in the present study is qualitatively consistent with this physical phenomenon: as the traverse speed rises from v = 0.75 mm/s to 1.75 mm/s, the thermal interaction time of the melt pool shrinks and there is no longer sufficient time for surface tension relaxation, leading to a gradual transition toward the Plateau–Rayleigh mode. The classification of all four points of the pure validation set as unstable shows that, for this process–electrode–material combination, the upper bound of the training range (v = 0.75 mm/s) forms a physical stability threshold in the parameter space.

This interpretation is also consistent with the internal flow signatures reported by Aucott et al. [10], who observed melt-pool flow behaviour in arc welding in situ using high-energy synchrotron radiation. Aucott et al. [10] showed that Marangoni convection can produce flow velocities in the range 0.1–0.5 m/s in the melt pool and that, when this flow velocity is balanced along the melt pool, it can trigger periodic width fluctuations. The width coefficient of variation (CV) and the linearity deviation (σ_cl_) quantified by image analysis in the present study are the quantitative signatures of this Marangoni–Plateau–Rayleigh interaction.

The selective training approach based on the quality filter (Section 2.5.3) ensures that the w_iz_ output is updated only with data derived from stable tracks; this prevents the w_iz_ value, which decreases in area on unstable tracks, from producing inconsistencies with the physics constraint. This approach falls within the “quality-aware loss configuration” category identified in the review by Faegh et al. [21]; it prevents noisy or out-of-regime data points from corrupting the training signal of the physics constraint.

### 4.5. Geometric Consistency Among Outputs and Physical Plausibility

The fact that the PINN model’s outputs on the pure validation set satisfy the two ordering constraints without exception indicates that physical consistency among outputs is largely preserved even outside the training range. The condition w_HAZ_ > w_iz_ (the heat-affected zone being wider than the melted track) is a direct reflection of the thermal-spread hierarchy defined by Cook and Murphy [11] in their analysis of laser melt-pool physics. The condition D_q_ > d_core_ (the arc core diameter being larger than the track core width) reflects the physical expectation that the footprint of the TIG arc plasma column on the powder-bed surface is wider than the melt pool. The third constraint (d_core_ ≤ w_iz_ ≤ 2.5·d_core_) was intended to bound the ratio of the two width definitions; as explained in Section 3.7, its lower bound as coded imposes an ordering opposite to the one the definitions produce and is not satisfied on the validation set, and its correction is identified in Section 5 as future work.

The preservation of these orderings outside the training range as well shows that the soft-constraint formulation of the geometric loss term L_geom_ steers the network weights toward a physically plausible parameter subspace. Khairallah et al. [1] showed in their high-resolution numerical models for LPBF that the flow-driven instability modes of melt-pool physics (denudation, keyhole instability, spatter) produce a consistent hierarchy in the geometric features of the track; the geometric constraints in the present study are a quantitative encoding of this hierarchy within the PINN framework.

Checking the partial derivatives of the model with respect to current, both ∂w_iz_/∂I and ∂d_core_/∂I remain positive across the training range and at the validation points. This is quantitative evidence that the monotonicity loss L_mono_ steers the network’s local rise–fall behaviour among training examples in a physically reasonable direction. This behaviour is consistent with Salzbrenner et al. [44]’s finding that a significant proportion of part-to-part variance in AM processes arises from local thermal-history fluctuations; the monotonicity constraint acts as a robust filter against noise among training examples.

### 4.6. Pure Speed Extrapolation: A Test of Physics-Informed Learning

Classical machine-learning evaluation approaches (k-fold cross-validation, leave-one-out, stacking, etc.) sample training and test sets from the same distribution and therefore measure only in-distribution generalisation capability. By contrast, extrapolation beyond the training range tests directly whether the model can produce physics-consistent predictions under out-of-distribution conditions. Zobeiry and Humfeld [19], in their physics-informed neural network solution of heat transfer equations for advanced manufacturing, showed that the network with engineered physics-informed features can capture the physics of the problem and make accurate predictions outside the training zone, whereas a network without physics features fails in this regime.

The pure speed extrapolation in the present study (four points extended to 1.33–2.33× the training upper bound) constitutes a quantitative test of this physics-informed learning claim in the PBAAM context. The fact that MAPE values for the three main outputs (D_q_, α_c_, w_HAZ_) remained below 10% shows that this claim holds even under small-sample conditions. Mozaffar et al. [45], in a graph-neural-network study of thermal modelling for metallic AM processes, emphasised that extrapolation performance is the most stringent test of the physical plausibility of a model; in this respect, the results of the present study support the practical applicability of the PINN-based process modelling approach for PBAAM.

On the other hand, the markedly lower extrapolation performance for d_core_ (MAPE 17.02%) shows that pure speed extrapolation is not equally successful for every output. This difference arises from the inability to consistently define d_core_ geometrically on unstable tracks; therefore, output-specific quality-aware interpretation is important in the extrapolation assessment.

## 5. Limitations

The proposed approach has several limitations. These limitations both reveal the boundaries of the method’s applicability and point to directions for future work.

Three limitations that bear on the interpretation of the results above are stated here before the individual subsections. First, no experimental condition was replicated, so the dataset does not support a direct estimate of experiment-to-experiment variance, and the reported errors cannot be separated from the repeatability of the process. Second, the pure validation set consists of four experiments that vary in a single direction at fixed current, work angle and working distance, and the corresponding target values are narrowly distributed. A set of this shape does not have the statistical power to separate competing models, and the classical regression baselines reported in Section 3.8 attain comparable errors on it without extrapolating at all. The extrapolation result should accordingly be read as evidence that the model does not fail under a modest out-of-distribution shift, rather than as a demonstration of general extrapolation capability. Third, the results reported in the main tables derive from a single random seed. The ablation of Section 3.8 was repeated with three seeds and the spread is substantial, with per-output standard deviations reaching 2.8 percentage points, so single-seed figures should be treated as one draw from a distribution rather than as the expected performance. Seed ensembles and Bayesian treatments of the network weights are the natural route to a calibrated predictive distribution and are identified here as future work, with the caveat that with four validation points any such calibration would itself be weakly determined.

A further item concerns the geometric loss. The lower bound of the third geometric constraint (g3) was found to be mis-specified relative to the definitions of the track core width and the areal track width (Section 3.7). The constraint is retained in its trained form so that the reported results correspond to the model actually trained, and its correction is identified here as future work.

Process phenomena that are not represented in the model also bear on the reliability of its predictions. Spatter ejects material from the melt pool and leaves the deposited track locally narrowed, and because the areal width used here is computed from the segmented track area it registers such losses as a reduction in width rather than as an outlier; the resulting variability is part of the coefficient of variation reported in Section 2.2 and is one reason why unstable tracks were excluded from the areal width target. Melt-pool fluctuation and the associated Plateau–Rayleigh instability produce the periodic width oscillation that the quality classification is designed to detect, and the model is deliberately not asked to predict the areal width for the conditions where that instability dominates. Instability of powder delivery affects the local bed density and therefore the effective thermal properties that the heat-conduction residual assumes to be uniform. This is not measured and enters the results as unmodelled scatter. Thermal accumulation is absent by construction, since each experiment is a single track on a fresh substrate, and it is precisely this term that would have to be added for multi-layer deposition. The common consequence of these four phenomena is that the achievable accuracy is bounded below by process variability rather than by model capacity, which is consistent with the observation in Section 3.10 that training error falls to near zero while validation error does not.

The uncertainty of the geometric measurements themselves is a further contribution that the reported errors do not separate. The features are extracted from segmented images at a scale set by an in-frame reference of known dimension, so the measurement chain comprises the calibration of that reference, the threshold that defines the track boundary and the operator-dependent choices in the segmentation. A relabelling of a random subsample in a second session was carried out as described in Section 2.3.4, but the agreement was not quantified, and no repeat measurements of the same specimen were made. The dataset therefore does not permit the measurement uncertainty to be separated from the process variability. Since supervised learning treats the labels as exact, any such uncertainty propagates directly into the fitted model and places a floor under the achievable validation error. A quantified repeatability study, in which a subset of specimens is measured independently by two operators and by an independent method such as profilometry, is required before the reported errors can be attributed to the model rather than to the measurement, and is identified here as a necessary step in future work.

### 5.1. Data Coverage and Sample Size

The training set consists of 36 experiments and the pure validation set of only 4 extrapolation points. The relatively wide bootstrap confidence intervals (for example, 0.91–8.49% for D_q_) are a direct reflection of this sample size. In future studies, a denser sampling of the parameter space (e.g., a central composite design rather than a full factorial) and extending the validation set to at least 10–15 points will narrow the confidence intervals. Sajadi et al. [13] examined in detail the effect of sample size on the stability of PINN performance in the online-learning regime.

### 5.2. Cross-Section Data Limitation

The penetration features (h_pen_, A_pen_) that could be extracted from cross-section (M-type) images were not used as PINN model outputs in this study. Metallographic preparation could only be applied to 16 experiments, and as most of these involved unstable tracks the penetration features could not be reliably included as model outputs. Katagiri et al. [9] showed in an LPBF single-track study that the estimation of penetration depth by finite element thermal analysis is critical for mapping the balling and lack-of-fusion process window. Addressing this gap for PBAAM will require systematic metallographic characterisation on all stable tracks.

### 5.3. Lack of Pure Validation for w_iz_

The classification of all four points of the pure validation set as unstable prevented the areal equivalent track width w_iz_ from being evaluated directly under pure speed extrapolation. This limitation can be addressed in future work by producing stable extrapolation points under different current, work angle and working distance conditions (for example, θ = 90°, d = 1.0 mm, higher current).

### 5.4. Operator Dependence in Manual Delineation

The manual delineation step (Section 2.3.2) contains an operator-dependent component; while reproducibility was ensured via re-labelling performed by a single operator, multi-operator consistency evaluation (inter-rater reliability) was not performed. Liu et al. [43] showed for interpretable machine-learning approaches in AM processes that the quality of operator-dependent data labelling directly affects prediction performance.

### 5.5. Single Material and Single Electrode Diameter

The study was carried out only for 316L stainless steel powder and a 1.6 mm WL20 electrode diameter. Çelikel and Balcı [7] showed, at a fixed 0.6 mm electrode diameter, that the electrode-to-powder-bed stand-off distance (0.3–0.8 mm) is a decisive parameter in the normalised energy framework; the effect of varying electrode diameter was not examined in the present study. The model’s generalisation capability for different electrode diameters and alternative metallic powders (Ti-6Al-4V, Inconel 718) should be examined in future studies, for which a transfer-learning approach is the natural route.

### 5.6. Single-Track Scale and Multi-Layer Transitions

This study is limited to the single-track scale; the sequential thermal history, inter-layer bonding and lack-of-fusion phenomena in multi-layer PBAAM processes are out of scope. Balcı and Alibeyoğlu [6] examined in detail the effect of overlap ratio and layer thickness on mechanical properties in five-layer PBAAM structures; adapting the present PINN model to multi-layer transitions will be a critical step for the practical industrial use of the system.

### 5.7. Computational Complexity, Data Quality Dependence and Transferability

Three further limitations should be stated. The model contains 13,767 parameters fitted to 36 conditions, a ratio of 382 parameters per sample. The consequence is visible in Section 3.10, where training error falls essentially to zero for four of the five outputs. The network has sufficient capacity to interpolate the training set exactly, and what prevents this from producing arbitrary behaviour between the training points is the combination of the physical constraints and the smoothness of the tanh activations rather than any capacity restriction. A smaller network would be preferable on statistical grounds, and the ablation of Section 3.8 indicates that much of the benefit comes from the shared representation rather than from depth. Second, the approach depends entirely on the quality of the experimental labels. The physical constraints restrict the space of admissible functions but cannot correct a systematic bias in the measurements, and the measurement uncertainty discussed above is not separable from the reported error. Third, transferability to other alloys is not demonstrated and should not be assumed. The material properties enter the heat-conduction residual through the product of effective density and melting enthalpy, which is alloy-specific, and the geometric ordering constraints depend on the wetting and spreading behaviour of the particular powder. A different alloy would require at a minimum a new set of property values and, more probably, retraining on new experiments. Whether a transfer-learning approach could reduce the number of experiments required is an open question that this study does not address.

## 6. Conclusions

In this study, a multi-modal physics-informed neural network (PINN) methodology was developed for simultaneously predicting the geometric features of single tracks of 316L stainless steel in the powder-bed arc additive manufacturing (PBAAM) process from four independent process parameters. The method systematically integrates a network architecture that joins five geometric features (D_q_, α_c_, w_HAZ_, d_core_, w_iz_) measured from two imaging modalities (top-view and arc) under a single shared trunk, with cross-section imaging retained for complementary verification; physics constraints including Rykalin-type heat-conduction scaling and the Ayrton voltage–current relation; and a composite quality score filter.

The main findings of the study can be summarised under the following headings.

First, the multi-modal PINN architecture displayed performance at the single-digit-percent MAPE level considered acceptable for engineering prediction in the PINN-AM literature for three main outputs in the pure speed extrapolation set: 4.25% for D_q_ (CI: 0.91–8.49), 6.29% for α_c_ (5.07–7.59), 8.06% for w_HAZ_ (6.08–9.82). The 17.02% MAPE for the track core width d_core_ reflects a limitation arising from the fact that d_core_ is not geometrically well-defined in the unstable track regime.

Second, the model’s Ayrton voltage sub-module converged to U(I) = 11.33 + 97.13/I through the physics loss term without any direct voltage measurement; this function is qualitatively consistent with the physical behaviour of low-current TIG arcs. This result constitutes a concrete application of the indirect parameter estimation capability of physics-informed learning in the metallic AM context.

Third, the definition of the areal equivalent track width (w_iz_ = A_iz_/L_iz_) provides a physically meaningful feature definition for single-track morphology by removing the systematic bias of the classical maximum-width definition on unstable and fragmented tracks. When combined with quality-aware loss formulation, this definition forms a methodological tool that prevents unstable tracks from corrupting the prediction signal.

Fourth, the composite quality score based classification labelled 23 of the 36 training experiments as stable and 13 as unstable; all four points of the pure validation set were labelled as unstable. The fact that the instability signature is characterised by a high agglomeration count (N_a_ = 12–53) and a comparatively high linearity deviation (σ_cl_ = 0.17–0.24 mm) supports the physical prediction that the Plateau–Rayleigh instability becomes dominant at speeds above the upper bound of the training range (v = 0.75 mm/s).

Fifth, the model’s outputs on the pure validation set satisfied without exception the two ordering constraints (w_HAZ_ > w_iz_; D_q_ > d_core_); the model’s partial derivatives with respect to current remained positive across the training range and at the validation points. The lower bound of the ratio constraint g3 was violated at all four validation points for the definitional reason explained in Section 3.7, and its correction is identified as future work. These results quantitatively show that the ordering constraints steered the network weights into a physically plausible parameter subspace.

Future studies should focus on three main axes. First, increasing the statistical confidence through denser sampling of the parameter space and extending the validation set. Second, adding the penetration features (h_pen_, A_pen_) to the PINN outputs via systematic metallographic characterisation from cross-section images. Third, generalising the model with a transfer-learning approach to different electrode diameters, alternative metallic powders (Ti-6Al-4V, Inconel 718) and multi-layer transitions. Progress along these three main axes will form the methodological infrastructure critical for the industrial applicability of the PBAAM process family.

## Figures and Tables

**Table 1:** Comparative summary of powder-bed and arc-based additive manufacturing processes. (—: not reported in the cited source).

Process	Heat Source	Feedstock	Current/Power	Speed	Layer	Material	Reference
LPBF	Laser	Powder bed	100–400 W	800–2000 mm/s	30–50 μm	316L, Ti-6Al-4V	[11]
EBM	Electron beam	Powder bed	60 mA	4000–8000 mm/s	50–100 μm	Ti-6Al-4V	[11]
WAAM	Arc (MIG/TIG)	Wire	100–300 A	5–20 mm/s	1–3 mm	Steel, Ti, Al	[27]
PAAM	Plasma arc	Wire	—	—	1–3 mm	Various	[4]
PPA-AM	Plasma transfer	Powder feed	80 A	5 mm/s	—	High-entropy	[2]
PBF-TIG	TIG arc	Powder bed	50–70 A	37–57 mm/min	~1.5 mm	H13 tool steel	[3]
TIG-PBF	TIG arc	Powder bed	—	—	—	Concept	[5]
PBAAM (This work)	Micro-TIG arc	Powder bed	15–25 A	0.25–0.75 mm/s	150 μm	316L	—

**Table 2:** Summary of the experimental matrix used in the PBAAM single-track experiments.

Set	I (A)	v (mm/s)	θ (°)	d (mm)	N
Training	{15, 20, 25}	{0.25, 0.50, 0.75}	{45, 90}	{0.6, 1.0}	36
Pure validation	25	{1.00, 1.25, 1.50, 1.75}	45	0.6	4
Total					40

**Table 3:** Physical residuals used in the loss function, with their basis, mathematical form, principal assumptions and the direction in which each constrains the network.

Term	Physical Basis	Form	Principal Assumptions	Constrains
*L*_phys_	Rykalin-type moving-source heat conduction	(*ŵ*_iz_ − *k*_w_*ŵ*_phys_)^2^, with *w*_phys_ ∝ √[*Q*_eff_/(*ρ*_eff_*H*_m_*v*)] and *Q*_eff_ = *ηU*(*I*)*I*·cos(90° − *θ*)/(1 + 0.15*d*)	Quasi-steady source; powder bed represented by effective bulk properties; melting energy taken as sensible heat to liquidus plus latent heat; constant arc efficiency η = 0.75; empirical attenuation with working distance	Track width scaling with energy input
*L*_geom_	Ordering entailed by the feature definitions	relu(*w*_iz_ − *w*_HAZ_ + 0.1)^2^ + relu(*d*_core_ − *D*_q_ + 0.1)^2^ + ratio terms	The heat-affected envelope is wider than the melted track; the arc envelope is wider than the arc core; the two width definitions remain within a bounded ratio	Consistency among outputs
*L*_mono_	Monotone rise in thermal input with current	relu(−∂*w*_iz_/∂*I*)^2^ + relu(−∂*d*_core_/∂*I*)^2^	Remaining parameters held fixed; monotonicity asserted only within the sampled range	Sign of the output derivatives
*L*_volt_	Admissible operating range of a low-current arc	relu(8 − *U*)^2^ + relu(*U* − 30)^2^ + relu(−*k*)^2^	Arc voltage between 8 V and 30 V; the Ayrton coefficient k is positive	Embedded sub-model parameters

**Table 4:** Material and thermophysical property values of 316L stainless steel used in the physics-based loss function.

Property	Symbol	Value	Source
Effective density of the powder bed	ρ_eff_	4235 kg m^−3^	Bulk density scaled by the measured packing fraction
Specific heat capacity	c_p_	500 J kg^−1^ K^−1^	Reference value for 316L, taken temperature-independent
Liquidus temperature	T_L_	1708 K	Reference value for 316L
Initial temperature	T_0_	298 K	Ambient, no substrate preheating
Latent heat of fusion	L_f_	260 kJ kg^−1^	Reference value for 316L
Melting enthalpy	H_m_	965 kJ kg^−1^	c_p_(T_L_ − T_0_) + L_f_
Arc efficiency	η	0.75	Representative value for TIG

**Table 5:** Composite quality score distribution and classification results.

Set	n	Stable (S ≥ 0.75)	Unstable (S < 0.75)	Score Range
Training	36	23	13	0.510–0.903
Pure validation	4	0	4	0.587–0.625
Total	40	23	17	0.510–0.903

**Table 6:** Prediction performance of the PINN model on the pure speed extrapolation set. MAPE and MAE values are reported as mean ± 95% bootstrap confidence interval (CI) (n: In experiment E39, the D_q_, α_c_ and d_core_ measurements were excluded due to boundary ambiguity in the corresponding source images; the sample size for these outputs is therefore 3.).

Output	n	MAPE Mean (%)	MAPE 95% CI (%)	MAE Mean	MAE 95% CI
D_q_ (mm)	3	4.25	[0.91, 8.49]	0.172	[0.036, 0.334]
α_c_ (°)	3	6.29	[5.07, 7.59]	2.98	[2.41, 3.66]
w_HAZ_ (mm)	4	8.06	[6.08, 9.82]	0.320	[0.239, 0.415]
d_core_ (mm)	3	17.02	[10.77, 21.62]	0.557	[0.322, 0.768]

**Table 7:** Values of the U(I) = 11.33 + 97.13/I function learned by the PINN voltage sub-module in the operating current range, compared with an indicative order-of-magnitude voltage range for short low-current argon TIG arcs, compiled from the qualitative behaviour reported for low-current arcs [22,41]; no voltage was measured in this study (Section 4.3). ✓: the learned voltage falls within the indicative range.

I (A)	Learned U(I) (V)	Indicative Range (V)	Within Range
15	17.81	15–20	✓
20	16.19	14–18	✓
25	15.22	13–17	✓

**Table 8:** Ablation of the framework components and comparison with classical regression models. Values are mean validation MAPE over the four measurable outputs; the neural configurations are averaged over three random seeds. n/a: not applicable; the classical regression models do not contain the shared trunk or the physics-loss components.

Configuration	Shared Trunk	Physics Terms	Validation MAPE (%)
A. Independent networks, data only	no	no	23.87
B. Independent networks, physics	no	partial	23.23
C. Shared trunk, data only	yes	no	12.51
D. Complete model	yes	yes	10.58
Ridge regression	n/a	n/a	8.83
k-nearest neighbours (k = 3)	n/a	n/a	9.81
Support vector regression (RBF)	n/a	n/a	9.16
Random forest	n/a	n/a	8.44
Gradient boosting	n/a	n/a	10.26

**Table 9:** Extended error metrics of the complete model on the training set and on the pure extrapolation validation set.

Output	Set	n	RMSE	MAE	MAPE (%)	R^2^
w_iz_	training	21	0.2565	0.2007	10.09	0.6227
w_HAZ_	training	36	0.0034	0.0021	0.05	0.9999
D_q_	training	36	0.0069	0.0044	0.09	0.9999
d_core_	training	36	0.0161	0.0104	0.38	0.9974
α_c_	training	36	0.0018	0.0012	0.003	1.0000
w_HAZ_	validation	4	0.3344	0.3226	8.06	−0.27
D_q_	validation	3	0.2114	0.1725	4.25	−0.94
d_core_	validation	3	0.5856	0.5477	17.02	−4.87
α_c_	validation	3	3.0217	2.9754	6.29	−12.53

**Table 10:** Relative influence of the process parameters on each predicted feature, from the mean absolute normalised partial derivatives of the trained model.

Output	Arc Current I	Traverse Speed v	Work Angle θ	Working Distance d
w_iz_	16.9	60.6	11.8	10.6
w_HAZ_	23.3	51.6	12.9	12.1
D_q_	19.9	53.2	15.7	11.3
d_core_	19.6	57.4	14.9	8.1
α_c_	19.6	58.1	11.3	11.0
Mean	19.9	56.2	13.3	10.6

## Data Availability

The dataset comprising the four process parameters and the five extracted features for all 40 experiments, together with the training script, is available from the corresponding author on reasonable request.

## Abbreviations

The following abbreviations are used in this manuscript:

AM	Additive Manufacturing
PBF	Powder-Bed Fusion
LPBF	Laser Powder Bed Fusion
EBM	Electron Beam Melting
WAAM	Wire Arc Additive Manufacturing
DED	Direct Energy Deposition
PAAM	Plasma Arc Additive Manufacturing
PPA-AM	Plasma Powder-fed Additive Manufacturing
PBF-TIG	Powder-Bed Fusion with Tungsten Inert Gas arc
PBAAM	Powder-Bed Arc Additive Manufacturing
TIG	Tungsten Inert Gas
CNC	Computer Numerical Control
HAZ	Heat-Affected Zone
CV	Coefficient of Variation
PINN	Physics-Informed Neural Network
PIML	Physics-Informed Machine Learning
Adam	Adaptive Moment Estimation
MAPE	Mean Absolute Percentage Error
MAE	Mean Absolute Error
CI	Confidence Interval
